# Environmental impact and sustainability of nanocellulose-based nitrated polymers in propellants

**DOI:** 10.1039/d5ra02169c

**Published:** 2025-07-10

**Authors:** Nurul Farhana Ahmad Aljafree, Mohd Nor Faiz Norrrahim, Alinda Samsuri, Wan Md Zin Wan Yunus

**Affiliations:** a Centre for Tropicalisation, Defence Research Institute, Universiti Pertahanan Nasional Malaysia Kem Perdana Sungai Besi 57000 Kuala Lumpur Malaysia farhana.aa@upnm.edu.my; b Research Centre for Chemical Defence, Defence Research Institute, Universiti Pertahanan Nasional Malaysia Kem Perdana Sungai Besi 57000 Kuala Lumpur Malaysia; c Department of Chemistry and Biology, Centre for Defence Foundation Studies, Universiti Pertahanan Nasional Malaysia Kem Perdana Sungai Besi 57000 Kuala Lumpur Malaysia; d Faculty of Defence Science and Technology, Universiti Pertahanan Nasional Malaysia Kem Perdana Sungai Besi 57000 Kuala Lumpur Malaysia wanmdzin@upnm.edu.my

## Abstract

Nanocellulose-based nitrated polymers derived from cellulose nanofibrils (CNFs), cellulose nanocrystals (CNCs), and bacterial nanocellulose (BNC), represent a significant innovation in the field of propellant materials. These renewable and biodegradable materials align with sustainability goals, offering reduced environmental impact compared to traditional synthetic propellants. This review highlights key findings on their environmental advantages, emphasizing greener synthesis methods, efficient production processes, and life cycle benefits. CNFs, CNCs, and BNC demonstrate competitive energetic properties while reducing reliance on non-renewable resources and minimizing harmful byproducts during production. Advances in enzymatic pretreatments, acid recovery systems, and renewable feedstocks have improved resource efficiency and scalability. Moreover, these nanocellulose-based nitrated polymers provide an opportunity to balance high-performance requirements with environmental priorities, addressing a critical challenge in modern propellant technology. Despite their promise, challenges such as performance optimization, regulatory compliance, and cost-effective scalability remain. This review calls for further research to develop safer nitration techniques, optimize production processes, and conduct comprehensive life cycle assessment (LCA). Collaborative efforts among researchers, industry, and policymakers are essential to overcome these barriers and establish nanocellulose-based nitrated polymers as sustainable alternatives for propellant applications.

## Abstract 2

Nanocellulose-based nitrated polymers derived from cellulose nanofibrils (CNF), cellulose nanocrystals (CNC), and bacterial cellulose (BNC), represent a significant innovation in the field of propellant materials.

## Graphical abstract

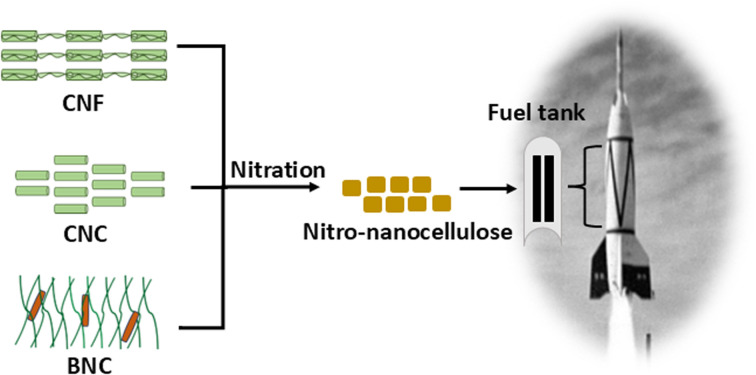

## 1. Introduction

Energetic materials encompass both explosives and propellants, with energetic compounds serving as the active chemical constituents that release stored energy during their operation.^1^ The first explosives in the form of fireworks were invented before 1000 CE.^2^ In 1249, the first explosive mixture of saltpeter, charcoal, and sulfur was conducted. Up until 1800, the advancement of explosives was primarily limited to the production and utilization of black powder. In the 19^th^ century, a surge in the study and development of high explosives, weapons technology, and propellants, led to the development of contemporary explosive technology.

On the other hand, propellants release their energy more gradually and have been a cornerstone of aerospace and defence technologies for decades, playing a crucial role in powering rockets, missiles, and various propulsion systems.^3^ Their development and optimization continue to be at the forefront of technological advancements in these fields. Recent research has focused on improving propellant performance, safety, and environmental impact. Propellant formulations contain several components, with the primary being an energetic material, commonly a nitro-containing organic chemical such as nitrocellulose (NC), nitroglycerin (NG), nitroguanidine (NQ), dinitrotoluene (DNT) often combined with other various perchlorate formulations.^1^

Among these, cellulose-based nitrated polymers, particularly NC, have traditionally been valuable for propellant technology owing to their high energy density and reliability in performance.^3^ NC have historically been a cornerstone of propellant formulations due to their high energy density, ease of production, and reliable performance.^4^ These polymers are derived from cellulose, a renewable natural resource, presenting an inherent advantage over fossil-fuel-based alternatives.

Nanocellulose shows strong potential by offering higher purity, tunable nanostructures, and high surface area.^5^ When nanocellulose is nitrated to form nanocellulose-based nitrated polymers it offers high-performance alternatives to conventional nitrocellulose. However, their production, usage, and disposal pose substantial environmental challenges that undermine their sustainability potential. Addressing these issues is crucial for aligning the benefits of nanocellulose-based nitrated polymers with modern sustainability goals.

Despite their renewable origins, the production and application of nanocellulose-based nitrated polymers are accompanied by significant environmental concerns. The nitration process, which involves treating nanocellulose with a mixture of nitric and sulfuric acids, generates hazardous byproducts such as nitrogen oxides and acid waste.^3^ Furthermore, the resource-intensive nature of the manufacturing process, including high energy requirements and the use of chemicals, diminishes the overall sustainability of these materials. The end-of-life disposal of these polymers, particularly in spent propellant forms, often results in residual nitrates that can contaminate soil and water systems.^6^ These challenges necessitate innovative solutions to mitigate their environmental impact while maintaining their functional performance.

Efforts to address these challenges have led to the exploration of various strategies aimed at improving the sustainability of nanocellulose-based nitrated polymers. One notable trend is the development of green nitration processes. Advances in green chemistry have enabled the use of alternative nitrating agents^7^ and the recycling of spent acids during synthesis,^8^ significantly reducing hazardous byproducts. Research has also focused on enhancing the biodegradability of nitrated cellulose through chemical modifications that allow for faster breakdown in environmental conditions without compromising the material's energy properties.^9^ Lifecycle optimization, involving comprehensive assessments of energy consumption, resource use, and waste generation, is increasingly being used to identify areas for improving sustainability across the entire production and usage chain.

Recycling technologies are emerging as a promising solution for reducing the environmental footprint of nanocellulose-based nitrated polymers.^10^ These techniques aim to recover and reuse nanocellulose and nitrate components from spent propellants, minimizing waste and environmental contamination. Furthermore, researchers are investigating hybrid materials that combine nanocellulose-based nitrated polymers with other biodegradable or eco-friendly materials, potentially improving their environmental compatibility while maintaining performance.

In this review, we will discuss the nanocellulose-based nitrated polymers as potential materials in propellants, the challenges posed by the environmental impact, sustainability of nanocellulose-based nitrated polymers, and evaluate the current solutions and trends aimed at enhancing their sustainability. By examining these advancements, the paper seeks to contribute to the ongoing efforts to develop eco-friendly propellant systems that align with the growing global emphasis on environmental conservation and resource efficiency.

### 1.1. Types of propellants

**Fig. 1:**
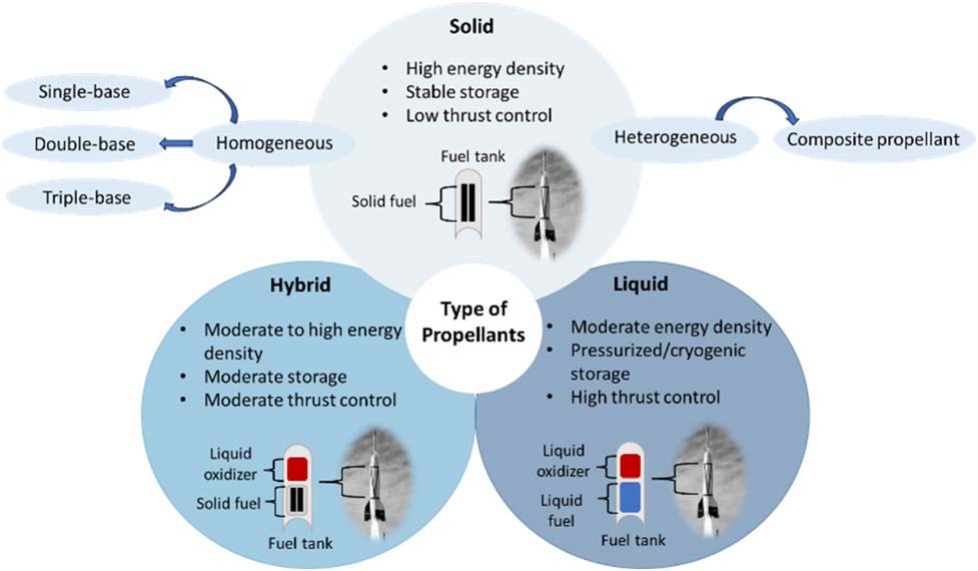
Classification of propellants and their applications.

Propellants are essential components in propulsion systems, used to generate thrust in applications ranging from aerospace and defence to fireworks and industrial devices.^11^ Propellants are most commonly categorized into three main types namely solid, liquid, and hybrid each with distinct properties and applications^12^ (Fig. 1). However, classification depends on context and specific criteria. For example, high-thrust rocket motors can be grouped into four categories based on the physical state of the propellants they carry: solid-propellant motors, liquid-propellant engines, gaseous-propellant engines, and hybrid-propellant engines.^13^

#### 1.1.1. Solid propellants

Solid propellants have been a cornerstone of aerospace and defence technologies for decades, playing a crucial role in powering rockets, missiles, and various propulsion systems. Their development and optimization continue to be at the forefront of technological advancements in these fields. Solid propellants are preferred in many applications due to their simplicity, reliability, long shelf life, and ability to provide high thrust-to-weight ratios^12^ A solid propellant comprises various chemical components, including an oxidizer, fuel, binder, plasticizer, curing agent, stabilizer, and cross-linking agent. The desired combustion characteristics for a specific mission determine the precise chemical composition.^14^ Based on their chemical composition and physical structure, propellants are typically classified as homogeneous or heterogeneous.^15^ In a homogeneous propellant, the ingredients are chemically linked and the resulting physical structure is uniform throughout. Homogeneous solid propellants are further classified into single-base, double-base, and composite types.^14^ Single-base propellants consist primarily of nitrocellulose and are known for their stability, making them suitable for small arms ammunition and older artillery systems.^3^ An example is nitrocellulose-based gunpowder. Double-base propellants combine nitrocellulose with nitroglycerin, enhancing energy density and burn control.^16^ For triple-base propellants, nitroguanidine can be added to a double-base composition.^15^ They are used in rockets, artillery, and small arms ammunition, with cordite being a notable example. Heterogeneous propellants consist of crystalline oxidizer particles bound within a polymeric fuel matrix. The commonly used oxidizers such as ammonium perchlorate and ammonium decompose thermally to yield high concentrations of oxygen, enhancing combustion performance. Composite propellants, on the other hand, use a polymer binder, such as hydroxyl-terminated polybutadiene (HTPB), mixed with a solid oxidizer like ammonium perchlorate.^17^ These propellants offer high thrust and are widely used in modern space launch vehicles, such as the Space Shuttle Solid Rocket Booster (SRB) fuel.

#### 1.1.2. Liquid propellants

Liquid propellants, consisting of separate liquid fuel and oxidizer components stored in tanks and combined in a combustion chamber, provide high specific impulse and allow for controlled thrust.^18^ This makes them ideal for spacecraft and launch vehicles. Liquid propellants are classified into cryogenic, hypergolic, and storable types. Cryogenic propellants, such as liquid hydrogen (LH_2_) and liquid oxygen (LOX), are stored at extremely low temperatures and are known for their high efficiency.^19^ They are commonly used in the upper stages of rockets, such as the Saturn V rocket's second and third stages. Hypergolic propellants ignite spontaneously upon contact with each other, providing reliability for applications requiring on-demand thrust, such as spacecraft thrusters.^20^ An example is the use of monomethylhydrazine (MMH) and nitrogen tetroxide (N_2_O_4_) in the Apollo Lunar Module.^21^ Storable propellants, like Aerozine 50 (a hydrazine derivative) and nitrogen tetroxide, are designed for long-term storage at ambient temperatures, making them suitable for military missiles and spacecraft with extended operational lifespans.

#### 1.1.3. Hybrid propellants

Hybrid propellants combine a solid fuel with a liquid or gaseous oxidizer, offering the safety and simplicity of solid propellants with the controllability of liquid systems.^22^ This combination is gaining interest for its reduced environmental impact and operational flexibility. A typical hybrid system uses a hydrocarbon-based solid fuel, such as HTPB, and a liquid oxidizer, such as liquid oxygen or nitrous oxide.^18^ SpaceShipOne, for instance, utilized a hybrid propellant system with HTPB as fuel and nitrous oxide as the oxidizer.^22^ Hybrid propellants are safer to handle than liquid propellants due to the separate storage of fuel and oxidizer. They also offer better thrust control compared to solid propellants, making them a promising choice for suborbital flights and small-scale missions.

Each type of propellant serves distinct purposes based on performance requirements and operational constraints. Solid propellants dominate in military and large-scale space applications, while liquid propellants are favored for their precision in spacecraft and rockets. Hybrid propellants, with their balance of safety, performance, and sustainability, are increasingly considered for emerging applications. Ongoing advancements in green chemistry and materials science are driving innovations across all categories, enabling the development of more efficient and environmentally sustainable propellants.

#### 1.1.4. Recent development on propellant application

**Fig. 2:**
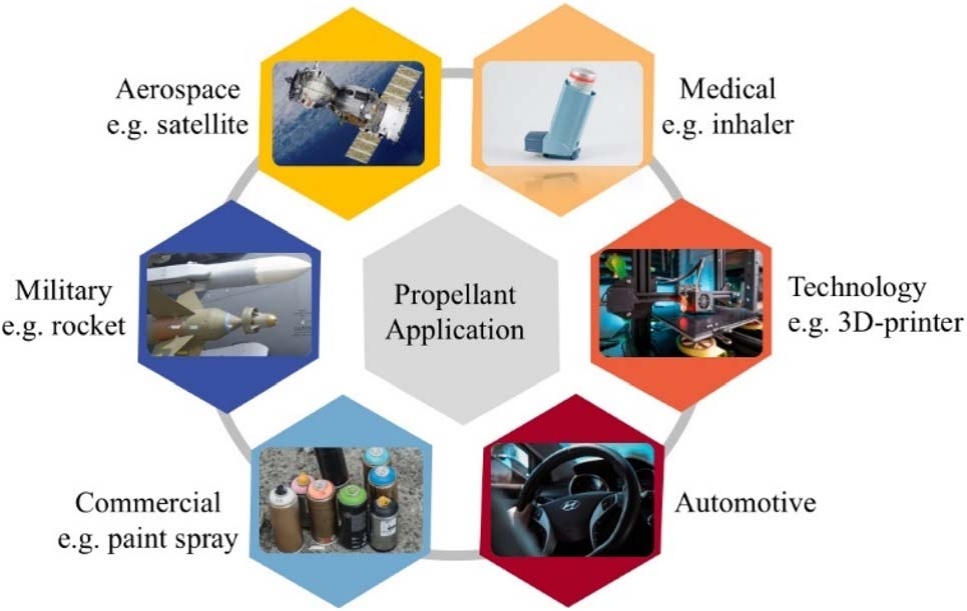
Propellant applications in modern industries.

Innovation are not only improving the efficiency and performance of propulsion systems but also addressing environmental concerns, cost-effectiveness, and safety. Recent developments span diverse applications, including military and commercial sectors, with each area benefiting from tailored propellant solutions (Fig. 2).

In military applications, the emphasis is on increasing the energy density, stability, and safety of propellants for missiles and ammunition. Enhanced solid propellant formulations, such as those incorporating nanomaterials, are being developed to improve burn rates and energy output. Nano-sized oxidizers and fuels, such as nano-aluminum particles, have been incorporated into composite propellants to achieve higher combustion efficiency and greater thrust.^23^ Additionally, the use of insensitive munitions, designed to minimize accidental detonation, is becoming standard in military-grade propellants to enhance safety during storage and transportation. Hybrid propellant systems are also being explored for tactical missile systems, combining solid and liquid components to enable adjustable thrust and increased mission versatility.

The commercial sector is witnessing the rise of hybrid and electric propulsion systems tailored for suborbital tourism, small satellite constellations, and low-cost launch services. Hybrid propulsion systems are gaining traction due to their safety, cost-effectiveness, and reduced environmental footprint. Meanwhile, electric propulsion systems, which rely on ion or Hall-effect thrusters, are becoming the preferred choice for small satellites due to their high efficiency and ability to operate for extended periods.^24^

Regulatory pressures and global environmental concerns are driving the adoption of greener alternatives in both chemical and electric propulsion systems. In the aerosol spray formulation, a propellant is rarely a single, standalone component; instead, mixtures of propellants are specially designed to meet certain requirements, like compatibility with products and packaging and, most crucially, container internal pressure.^25^ The physical and pharmacological characteristics of the propellant and drugs in pressurised metered dose inhalers (pMDI), provide a scientific reason for the increasing application of pMDIs and valved holding chambers (VHC) in managing acute severe asthma. For instance, hydrofluoroalkane such as HFA-134a and HFA-227 were used to aerosolize the medication by rapidly vaporizing and dispersing it into a fine mist for inhalation.^26^ In addition, bio-based fuels derived from renewable resources are being studied as potential replacements for conventional propellant components. Researchers are also exploring ways to recycle spent propellants and reduce the ecological impact of residual byproducts. The integration of additive manufacturing (3D printing) in propellant production is another breakthrough, enabling the precise fabrication of complex geometries for combustion chambers and nozzles, thereby improving performance and reducing waste.^27^

Lastly, advancements in smart and adaptive propulsion systems are revolutionizing the way propellants are used. Systems equipped with real-time monitoring and control capabilities can dynamically adjust combustion parameters, optimizing fuel efficiency and performance during flight. These innovations are particularly valuable in reusable and multi-mission platforms, where adaptability is critical to mission success.

The field of propellant technology is undergoing rapid innovation across all sectors. From green alternatives for satellite propulsion to high-performance propellants for space exploration and defence, recent developments are addressing both technical and environmental challenges. These advancements underscore the importance of interdisciplinary research and collaboration in shaping the future of propulsion systems.

### 1.2. Overview of nitrated polymers in propellant formulations

Nitrated polymers, particularly cellulose-based nitrated compounds like nitrocellulose, have been foundational components in propellant formulations for over a century. These polymers are characterized by their high energy content, rapid combustion rates, and flammability under a wide range of conditions, making them indispensable in various applications, including military, aerospace, and industrial sectors.^28^ NC, derived from the nitration of cellulose, is the most common example of a nitrated polymer used in propellants.^29^ Its renewable origin from plant-based cellulose and its ability to undergo controlled nitration make it a versatile and reliable ingredient in both solid and hybrid propellant systems.

In propellant formulations, nitrated polymers typically serve as binders, fuels, or energetic additives, depending on the type of propellant (Table 1). In single-base propellants, NC is the sole energetic component, delivering both structural integrity and the required energy output.^30^ In double-base formulations, NC is combined with NG to enhance energy density and burn characteristics, resulting in higher thrust capabilities.^31^ Beyond their role in traditional solid propellants, NCs are also employed in composite formulations, where they act as energetic binders that contribute to the overall combustion process. Their ability to impart mechanical strength and thermal stability to the propellant matrix is a critical factor in achieving reliable performance under extreme conditions.

**Table 1:** Comparison of nitrocellulose, nitroglycerin, and nitramines in propellant formulations

Property	Nitrocellulose (NC)	Nitroglycerin (NG)	Nitramines (*e.g.*, RDX, HMX)
Chemical structure	Nitrated cellulose polymer	Nitrated glycerol ester	Nitrated cyclic amine compounds
Energy density (MJ kg^−1^)	∼3.7	∼6.3	∼5.4 (RDX)
			∼5.7 (HMX)
Stability	Sensitive to thermal decomposition	Highly sensitive to shock and temperature fluctuations	Less sensitive than NG
Burn rate	Moderate (0.5–20 mm s^−1^)	Fast (20–100 mm s^−1^)	Very fast (>100 mm s^−1^)
Applications	Single-base propellants (small arms, fireworks)	Double-base propellants (rockets, missiles)	Composite propellants
	Double-base propellants	Triple-base propellants	High-performance explosives
Advantages	Cost-effective	High energy output	Extremely high energy
	Good mechanical properties	Enhances burn rate in propellant formulations	Thermal stability
	Easy to process		
Disadvantages	Limited energy density	High sensitivity to shock and heat	Prone to hydrolysis
	Susceptible to degradation over time	Toxic and hazardous	Complex synthesis and handling
Typical use cases	Small-caliber ammunition	Rocket motors	Advanced missile systems
	Pyrotechnics	Military-grade explosives	Space launch vehicles
	Industrial blasting		
Production challenges	Requires large-scale nitration of cellulose, which involves hazardous acids	Highly dangerous production process due to NG's sensitivity	Complex and costly synthesis
	Energy-intensive process	Requires strict safety protocols	Involves toxic intermediates and byproducts
Usage challenges	Degrades over time, requiring stabilizers	High risk of accidental detonation during handling and use	High sensitivity
	Limited performance in high-energy applications	Toxic fumes during combustion	Limited lifetime
			Lower detonation energies
Disposal challenges	Decomposition releases harmful gases (*e.g.*, NO_*x*_)	Toxic and hazardous waste	Persistent environmental pollutants
	Requires controlled incineration or chemical neutralization	Difficult to safely dispose of due to sensitivity	Requires specialized disposal methods to avoid contamination
Environmental impact	Production and disposal contribute to air and water pollution	High environmental toxicity	Toxic residues persist in soil and water
	Photooxidation	Production byproducts are hazardous to ecosystems	High carbon footprint due to complex synthesis
Sustainability concerns	Accumulation of waste	Unsustainable due to high toxicity and safety risks	Persistent in the environment
	Environmental hazards during production and disposal	Poor environmental footprint	Limited potential for sustainable production
References	3, 29 and 32	3, 33 and 34	35 and 36–38

By controlling the degree of nitration, manufacturers can adjust the energy output, burn rate, and sensitivity of the polymer.^3^ For instance, highly nitrated forms of nitrocellulose are used in applications requiring rapid energy release, such as artillery and rocket propellants, while less nitrated forms are employed in applications requiring slower and more controlled combustion.^39^ This flexibility has made nitrated polymers a key material in diverse propellant formulations, from small-caliber ammunition to large-scale missile systems.

Despite their advantages, the use of nitrated polymers in propellants is not without challenges. The production process involves hazardous chemicals, including concentrated nitric and sulfuric acids, leading to environmental and safety concerns.^40^ The resulting byproducts, such as nitrogen oxides and acid waste, require careful management to prevent ecological damage. Furthermore, nitrated polymers exhibit limited biodegradability, raising concerns about their long-term environmental impact, especially in military training and operational environments where residue accumulation can occur.

Recent advancements in nitrated polymer technology aim to address these issues while enhancing performance. Innovations such as green nitration techniques, which reduce hazardous byproduct generation, and hybrid formulations incorporating biodegradable or eco-friendly additives are gaining traction.^30^ Additionally, researchers are exploring modifications to the molecular structure of nitrated polymers to improve their energy efficiency and compatibility with next-generation propellant systems. The integration of nitrated polymers with emerging technologies, such as nanomaterials and additive manufacturing, further highlights their evolving role in modern propellant formulations.

The nitrated polymers are integral to the field of propellant technology, offering unmatched energy performance and adaptability. While their traditional applications remain critical, ongoing research and development are focused on addressing environmental and safety concerns, ensuring that these materials continue to meet the demands of modern propulsion systems.

### 1.3. The environmental impact and sustainability of nitrated polymers in propellants

The growing global emphasis on environmental conservation and sustainability has brought the ecological impact of traditional materials, including nitrated polymers in propellants, into sharp focus. Their production, usage, and disposal present significant environmental challenges that must be addressed to align with evolving sustainability goals.^3^ Assessing and mitigating the environmental impact of these materials is crucial to ensure their continued utility while minimizing harm to ecosystems and human health.

The production of nitrated polymers is inherently resource-intensive, involving the use of concentrated nitric and sulfuric acids in the nitration process.^41^ Furthermore, the energy-intensive nature of the manufacturing process contributes to greenhouse gas emissions, amplifying the environmental footprint of these materials.^32^ In addition to production challenges, the degradation of nitrated polymers in the environment is slow, resulting in long-term persistence and potential accumulation in ecosystems. This is due to their chemical structure, which makes them resistant to biodegradation. The presence of nitrate groups (–O–NO_2_) major contributor to its instability and has a relatively low bond energy, but also less prone to breaking down by natural processes, especially in environments where microorganisms that can degrade such compounds are limited.^42^ This is particularly concerning in military training zones and other areas where spent propellants may be deposited over time.

Beyond production and disposal, the operational use of nitrated polymers poses environmental risks.^43^ Although nitrated polymers, such as NC and NG, are widely used in propellant, the combustion of these materials produces residues, including nitrate compounds, nitrogen oxides (NO_*x*_), and carbon-based byproducts.^3^ For instance, NC decomposes exothermically, releasing gases like nitrogen (N_2_), carbon monoxide (CO), carbon dioxide (CO_2_), and water vapor (H_2_O), along with solid residues. The nitro groups (–NO_2_) in NC break down to form nitrogen oxides (NO_*x*_), while incomplete combustion can leave behind unreacted nitrates and carbonaceous particles. Similarly, NG combustion produces NO_*x*_ and other byproducts due to the breakdown of its nitrate ester bonds.^44^ Additives and stabilizers in propellant formulations can further contribute to residue formation, as can trace impurities in the raw materials. Nitrate residues resulting from the burning of nitrated polymers can adversely affect the environment, leading to problems such as water contamination if the nitrates infiltrate water sources.^45^ The potential for such impacts highlights the urgent need for comprehensive environmental assessments across the entire lifecycle of nitrated polymers, from raw material extraction to end-of-life disposal. Without such assessments, the cumulative ecological damage may undermine the long-term sustainability of these materials in propellant applications.

Recognizing these challenges, the scientific community has increasingly prioritized the development of green alternatives and the integration of lifecycle analysis (LCA) in the evaluation of nitrated polymers. LCA provides a comprehensive framework for assessing the environmental impact of these materials by accounting for energy consumption, emissions, waste generation, and ecological risks across their entire lifecycle.^46^ This approach enables researchers and manufacturers to identify key areas for improvement, such as optimizing production methods, enhancing biodegradability, or recycling spent materials.

## 2. Synthesis and energetic property of treated nanocellulose polymers as potential material in propellants

The traditional NC has long been a cornerstone in propellant formulations owing to its high energy content and favorable combustion properties. However, recent advancements in cellulose nanotechnology, cellulose nanofibrils (CNFs), cellulose nanocrystals (CNCs), and bacterial nanocellulose (BNC) represent advanced forms of cellulose that have garnered attention due to their nanoscale structures and exceptional mechanical and thermal properties.^47^ By integrating these nanocellulose derivatives into propellant systems, researchers aim to achieve improved energy output, better stability, and a more sustainable alternative to conventional petrochemical-based propellants (Table 2). Among these, CNFs, CNCs, and BNC offer unique advantages which allow for efficient functionalization and integration into composite matrices offering superior structural integrity and chemical compatibility for nitration.

**Table 2:** Physical and chemical characteristics of nanocellulose

Property scale	Cellulose nanofibrils (CNFs)	Cellulose nanocrystals (CNCs)	Bacterial nanocellulose (BNC)
Source	Wood, cotton, agricultural residues, waste biomass	Wood, agricultural residues, energy crops	Microbial fermentation (*e.g.*, *Komagataeibacter xylinus*)
Structure	Fibrillar, high surface area, flexible	Rod-like, crystalline, high crystallinity	Three-dimensional nanofiber network, highly pure
Biodegradability	High, decomposes into non-toxic byproducts	High, naturally biodegrades	High, fully biodegradable under natural conditions
Energy density	High, tunable for energy release, efficient nitration	High, optimized for efficient nitration	High, exceptional for energetic material applications
Mechanical properties	Flexible, strong, can be used for reinforcement in composites	Rigid, strong, enhances mechanical strength	High tensile strength, exceptional mechanical properties
Surface area	Large, facilitates high nitration and functionalization	Moderate, efficient for crystalline structure optimization	Extremely high, ideal for high-efficiency nitration
Production process	Delignification, TEMPO oxidation, mechanical fibrillation	Acid hydrolysis, acid concentration control	Bacterial fermentation, simple post-processing
Sustainability	Derived from renewable, waste-based biomass, minimal waste generation	Derived from renewable biomass, uses sustainable extraction methods	Produced through waste valorization, utilizes non-food biomass
Energy consumption (production)	Moderate, energy-efficient methods (*e.g.*, energy-efficient homogenization)	High, energy-intensive acid hydrolysis and purification processes	Moderate to high, energy-intensive fermentation and drying
Environmental impact	Low, biodegradable, reduces long-term pollution	Moderate, can be energy-intensive but sustainable when sourced responsibly	Very low, minimal ecological impact, water and energy consumption during fermentation
Nitration efficiency	Highly efficient nitration, adaptable for high energy output	High nitration efficiency, energy density comparable to conventional materials	High, customizable nitration, high purity and performance
Toxic byproducts (during synthesis)	Low, green chemistry advances reduce solvent and waste use	Moderate, acid waste disposal concerns	Low, microbial process avoids toxic chemicals
Carbon footprint	Low, especially with the integration of renewable energy in production	Moderate, reduces reliance on fossil fuels when using waste biomass	Very low, promotes carbon neutrality through sustainable processes
Applications in propellants	High-performance, flexible propellants, composite matrices	High-energy propellants, coatings, films, and additives	High-performance, biodegradable propellants, sustainable formulations
References	48, 49 and 50	8, 9 and 51	52–54

### 2.1. Cellulose nanofibrils (CNFs)

The production phase of CNFs for propellant applications involves the raw cellulose undergoes a delignification-bleaching process to remove non-cellulosic components.^55^ This is often followed by TEMPO-mediated oxidation, which facilitates subsequent nano-fibrillation by introducing carboxyl groups on the cellulose surface.^48^ The oxidized cellulose is then subjected to mechanical treatment, typically using high-pressure homogenization, to break down the fibers into nanoscale dimensions.^49^ Alternative methods, such as stone grinding or ball milling, have also been explored to reduce energy consumption during the fibrillation process.^56^

CNFs are generated from renewable biomass, which in turn reduces dependence on finite fossil resources and is consistent with global initiatives to create more environmentally friendly technologies. The source of raw materials for the manufacturing of CNF has shifted more toward lignocellulosic waste streams, which is consistent with the circular economy and sustainability objectives.^50^ Agricultural residues, such as sugarcane bagasse, windmill palm, and almond stems, have been successfully used to produce CNF.^57^ These waste materials offer several advantages, including reduced feedstock costs and minimized environmental impact compared to primary cellulose sources. The selection of raw materials for CNF production takes factors such as cellulose content, lignin content, and fiber morphology.^58^ The sustainability of lignocellulosic waste streams is assessed in terms of environmental and economic implications, along with chemical and physical properties.^50^

Integrating renewable energy sources into CNF production facilities has emerged as a viable strategy to mitigate the environmental impact. Additionally, advancements in equipment design, such as energy-efficient homogenizers and innovative shearing devices, are critical in minimizing energy demands.^59^ LCA of CNF production reveal that energy-efficient processes and renewable energy integration significantly enhance the overall sustainability of CNF as a raw material for propellant applications.^60^.

CNFs' excellent mechanical properties and tunable nanostructure allow for optimized performance in propellants, achieving comparable or superior energy output while being derived from a sustainable, renewable resource.^9^ Thus, cellulose nanofibrils present an opportunity to bridge high-performance energetic materials with environmentally conscious solutions, contributing to cleaner, more sustainable technologies in defense, aerospace, and industrial applications.

### 2.2. Cellulose nanocrystals (CNCs)

CNCs have garnered interest as a sustainable and eco-friendly precursor for nanocellulose-based nitrated polymers in propellant formulations, alongside CNFs. CNCs are rod-like, highly crystalline nanoparticles extracted from natural cellulose sources through acid hydrolysis.^51^ Their high crystallinity, uniform size distribution, and exceptional mechanical properties^61^ make them particularly well-suited for nitration, enabling the production of highly energetic materials like NC. The production phase of CNCs plays a critical role in determining the environmental footprint and overall sustainability of these materials. The initial step involves extracting cellulose from renewable plant sources, such as wood, agricultural residues, or dedicated energy crops.^9^ As cellulose is one of the most abundant biopolymers, the raw material sourcing phase is inherently more sustainable compared to traditional synthetic polymers.

CNCs are isolated by acid hydrolysis, where concentrated sulfuric or hydrochloric acid is used to break down the cellulose into crystalline segments.^51^ While this method produces high-quality CNCs, it raises concerns regarding the disposal of acids and the energy-intensive nature of the process. Some studies have explored alternative, more sustainable approaches, such as enzymatic hydrolysis or the use of milder acids, which reduce the environmental load associated with CNC production.^9^ Moreover, research into greener processes, including the use of ionic liquids or supercritical CO_2_, holds promise for mitigating the negative environmental impacts while enhancing the yield and properties of CNCs.^8^

Energy consumption during the synthesis of CNCs and their nitration for propellant applications typically carried out by using a mixture of concentrated nitric and sulfuric acids, requires careful control of temperature and reaction conditions to achieve the desired polymer properties.^62^ This process is often energy-intensive and can result in the release of hazardous by-products. Alternative nitration methods that are energy-efficient and produce fewer pollutants are an active area of research.

### 2.3. Bacterial nanocellulose (BNC)

The production phase of BNC is distinct from traditional plant-based nanocellulose, as it involves the fermentation of sugars by certain bacteria, such as *Acetobacter xylinum* and *Gluconacetobacter* species.^52^ BNC synthesized by specific strains of bacteria such as *Komagataeibacter xylinus*, is an emerging and highly sustainable material for producing nanocellulose-based nitrated polymers in propellants.^63^ For instance, BNC was biosynthesized using three microbial producers: two individual strains, *Komagataeibacter xylinus* B-12429 and *Komagataeibacter xylinus* B-12431, and a symbiotic culture, *Medusomyces gisevii* Sa-12.^63^ The symbiotic culture demonstrated a significantly higher BNC yield, surpassing that of the individual strains by 44–65%. The physicochemical properties of BNC, such as nanofibril width, degree of polymerization, elastic modulus, Iα allomorph content, and crystallinity index, were influenced by the type of microbial producer rather than the composition of the nutrient medium.^64^ This highlights the critical role of microbial selection in tailoring BNC properties for specific applications.

BNC offered a unique combination of purity, nanostructural precision, and renewability.^64^ BNC production offers significant advantages over plant cellulose, primarily due to its high degree of polymerization and lack of lignin, which reduces the need for complex processing steps like delignification.^53^ Furthermore, BNC can be cultivated in various settings, including waste-streams from agricultural or industrial processes, contributing to circular bioeconomy models.^54^ The raw material sourcing for BNC production generally revolves around the use of simple, renewable sugars, such as glucose or fructose, which can be sourced from a variety of agricultural by-products, food waste, or even industrial waste streams.^65^ Choosing cost-effective culture media aligns with the zero-waste economy trend, emphasizing the repurposing of agro-industrial waste for producing biopolymers like BNC. Several studies explore other waste sources, such as grape marc and potato waste, for BNC production.^66^ Grape marc, though limited in monosaccharides post-fermentation, can be acid-hydrolyzed to enhance fermentable sugar content, while potato juice, requiring minimal pretreatment, yields BNC comparable to commercial media in both quality and yield.^66^ These approaches not only reduce production costs but also support a circular bio-economy by transforming waste into valuable biopolymers, advancing sustainable industrial practices.

When sourced from agricultural residues or non-food biomass, BNC production can become a highly sustainable process. For instance, a study was conducted to optimize BNC production using eucalyptus bark hydrolysate (EBH) as a sustainable carbon source, achieving a 39.7-fold increase in yield (8.29 ± 0.21 g L^−1^) compared to standard media.^54^ However, fermentation wastewater (WaF) and combined washing streams (WaW) exhibited high organic loads, requiring treatment. Anaerobic digestion effectively treated the wastewater, achieving over 87% mechanization and methane yields of 486–544 L per kg volatile solids, though aerobic treatment failed to further reduce COD levels.^54^ Remarkably, treated wastewater was recycled into production at 45% without compromising BNC yield. This work demonstrated a sustainable, cost-effective approach to BNC production and wastewater management, advancing its industrial viability.

The synthesis processes of BNC, while relatively straightforward in terms of bacterial fermentation, still present challenges related to scaling and maintaining high yields. The fermentation process itself is energy-intensive, requiring controlled temperature, pH, and aeration for optimal bacterial growth and cellulose production.^64^ The primary methods for BNC production, including static culture, agitated culture, fed-batch culture, and bioreactors, critically influence the material's structural and chemical properties, thereby determining their suitability for specific applications.^67^ For instance, fed-batch fermentation using hydrolyzed paper sludge or glycerol as carbon sources increased BNC yields by 2.1–4.6-fold compared to batch methods.^68^ Similarly, intermittent feeding with alcohol lees achieved a BNC yield of 4.4 g L^−1^, doubling that of static cultures.^69^ Advanced bioreactors, such as rotating biological contactors (RBCs) and airlift systems, further improve production efficiency by enhancing oxygen transfer and reducing operational costs. RBCs, for example, increased BNC wet and dry weights by 64% and 47%, respectively, under optimal conditions. Additionally, utilizing agro-industrial waste like grape bagasse and potato residues as cost-effective media, combined with acid hydrolysis to boost fermentable sugars and phenolic content, supports sustainable and scalable BNC production.^66^ These innovations highlight the potential for industrial-scale BNC synthesis while aligning with circular economy principles.

**Fig. 3:**
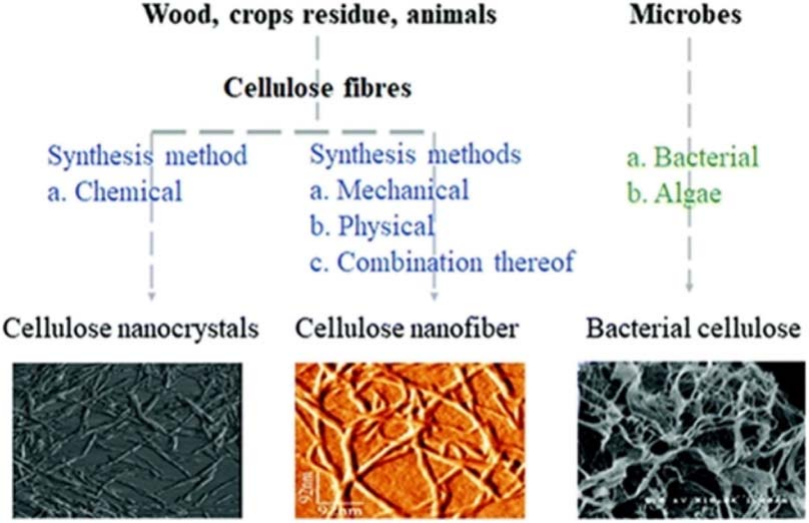
Morphology of CNCs, CNFs, and BNC. Reproduced from ref. 47 with permission from the Royal Society of Chemistry.

One significant environmental advantage of BNC is its minimal ecological impact during both production and disposal. Unlike traditional cellulose sources, BNC does not require extensive land use, deforestation, or chemical-intensive extraction processes.^70^ Its cultivation in controlled bioreactors reduces resource consumption, such as water and energy, and eliminates the need for harmful pesticides or fertilizers. Together with CNFs and CNCs, BNC forms a trio of renewable, high-performance precursors for nanocellulose-based nitrated polymers. As a renewable, biodegradable, and high-purity material, BNC demonstrates the potential to revolutionize propellant formulations, aligning advanced energetic technologies with the principles of sustainability and environmental stewardship (Fig. 3).

Despite CNCs, CNFs, and BNC extensive applications in composites, coatings, and biomedical fields, their chemical modification through nitration remains underexplored. The introduction of nitrate (–NO_3_) functional groups can impart unique properties such as enhanced thermal reactivity, energy density, and solubility in organic solvents, making these materials promising for advanced applications, particularly in energetic materials.^29^

### 2.4. Energetic performance of nanocellulose-based nitrated polymers

#### 2.4.1. Detonation parameter

Recent experimental work on high energy density formulations has demonstrated that combining hydrazine 3-nitro-1,2,4-triazol-5-one/ammonium nitrate (HNTO/AN) cocrystals with nitrocellulose (NC) or nanostructured microcrystalline cellulose nitrate (NMCC) delivers detonation velocities between 7955–8062 m s^−1^ at 60 : 40 wt% composition that significantly outperforming traditional energetic materials like nitroglycerin (7700 m s^−1^) and triaminotrinitrobenzene (TATB, 7940 m s^−1^).^71^ In formulations integrating nanocellulose-based binders with plasticizers and nitramines, nanostructured bacterial cellulose nitrate (NBC) notably enhances impact resistance and burning rate without sacrificing other key properties.^72^ Heat of detonation measurements further validated the energetic advantages of nanocellulose-based systems. Pure NC/NG propellants exhibited a heat release of approximately 4873.7 J g^−1^, whereas adding NBC boosts performance to 4879.7 J g^−1^ (NC/NBC–NG) and slightly modulates calorific output in mixed formulations (NC/NBC–NG/DEGDN = 4833.5 J g^−1^; NC/NBC–DEGDN = 4476.3 J g^−1^; NC/NBC alone = 4504.0 J g^−1^).^72^ These values indicate that incorporating NBC does not compromise energetic output and can even enhance it in selected blends. NMCC and NBC demonstrated exceptional energetic performance, including detonation velocities on par with or exceeding standard nitrate esters (NC, NG, TATB) and improved mechanical and combustion characteristics.

#### 2.4.2. Thermal behaviour

Thermogravimetric analysis (TGA) of NC and nanostructured microcrystalline cellulose nitrate (NMCC) shows a single major mass-loss stage (≥95%) at 205.9 °C for NC and 198.7 °C for NMCC, whereas the hydrazine 3-nitro-1,2,4-triazol-5-one/ammonium nitrate (HNTO/AN) salt decomposes in two steps (195–235 °C) with ∼95% weight loss.^71^ In another study, energetic binder nitrated bacterial cellulose (NBC) as the gel matrix was incorporated with CL-20 (hexanitrohexaazaisowurtzitan).^4^ The onset of decomposition in NBC–CL-20 occurs at lower temperatures (∼145 °C) compared to raw NBC (∼185 °C) and CL-20 (∼244 °C). This was due to the nano-scale particle size facilitating heat and mass transfer that leads to the acceleration of the decomposition process. Additionally, native cellulose and cellulose microcrystals nitrates (NCN and CMCN) from the same bioresource exhibit a single endothermic decomposition. Non-isothermal decomposition kinetics, analyzed *via* isoconversional integral methods, showed that ANCN and ACMCN exhibit strong thermal stability with an autocatalytic decomposition mechanism and marginally lower Arrhenius parameters compared to standard NCN.^73^ The thermal profiles indicated that all samples display a single endothermic decomposition peak at distinct temperatures: 317.3 °C for NC, 354.1 °C for CMC, 312.4 °C for ANCN, and 351.2 °C for ACMCN.

#### 2.4.3. Enthalpy of decomposition

Non-isothermal kinetic analysis using isoconversional integral methods revealed that the energetic composites NC@HNTO/AN and NMCC@HNTO/AN (60 : 40 wt%) exhibit average activation energies (*E*_a_) of 128 kJ mol^−1^ and 124 kJ mol^−1^, respectively which both significantly lower than those of pure NC (155 kJ mol^−1^) and NMCC (140 kJ mol^−1^).^71^ The mean values of *E*_a_ during decomposition for NC@HNTO/AN and NMCC@HNTO/AN are 121 kJ mol^−1^ and 101 kJ mol^−1^, respectively, compared to the HNTO/AN cocrystal alone at 121 kJ mol^−1^. These reductions indicate that nitrate-ester cellulosic polymers decrease both the reactivity and decomposition rate of the HNTO/AN component. The NMCC composite also delivers a higher overall enthalpy of decomposition, 1697.1 J g^−1^*versus* 1415.8 J g^−1^ for the NC composite which demonstrated superior energetic output.^71^

Further evidence of enhanced performance is seen in NBC/CL-20 composites, which release substantial heat during decomposition (3346 J g^−1^), considerably more than both the NBC/CL-20 physical mixture (2394 J g^−1^) and the sum of individual components (NBC 668.2 J g^−1^ + CL-20 1498 J g^−1^ ≈ 2166.2 J g^−1^), confirming a synergistic effect from the nanocellulose matrix.^4^ In terms of combustion performance, NBC/NC nitramine gun propellants show higher burning rates under identical environmental pressures: at 100 MPa, rates rise from 9.70 m s^−1^ (NC) to 10.10 m s^−1^ (NBC/NC), and at 200 MPa from 18.81 m s^−1^ to 19.93 m s^−1^, attributed to improved thermal conduction and combustion facilitated by the NBC network structure.^72^

Finally, among various nanocellulose derivatives tested, granules with the highest crystallinity index (G(CI)_3_) exhibited the greatest decomposition enthalpy (2246 J g^−1^), and increased nitrogen content in NC corresponded to higher heat of combustion, rising from 3079 J g^−1^ to 3630 J g^−1^.^74^ Moreover, EDA-modified nitrated celluloses released more energy than unmodified counterparts in descending order: ACMCN (−4475 kJ mol^−1^) > ANCN (−4336 kJ mol^−1^) > CMCN (−2793 kJ mol^−1^) > NCN (−2672 kJ mol^−1^).^73^ This clearly shows that incorporating nitrogen-rich functional groups alongside nitrate esters in the same polymer backbone markedly enhances combustion heat.

Nanocellulose-based nitrated polymers demonstrate significant enhancements in energetic performance compared to traditional nitrocellulose. In this context, nanocellulose-based nitrated polymers can surpass nitrocellulose, nitroglycerin, and nitramine due to their unique characteristics, including higher surface area and tunable properties, making them advantageous for applications in energetic materials, composites, and drug delivery systems. The nanoscale dimensions of nanocellulose yield a significantly higher surface area, which enhances reactivity and promotes more efficient interactions with additives or oxidizers resulting in faster combustion kinetics and improved energetic performance.^75^ In contrast, traditional NC, although energy-dense, lacks this level of reactivity due to its larger particle structure.

Chemical modifications, including surface functionalization, enable precise control over hydrophobicity, reactivity, and mechanical strength.^76^ For instance, during thermal analyses, materials like nitrated bacterial cellulose (NBC) and nitrate-glycerol-ether cellulose (NGEC) demonstrate higher decomposition activation energies and superior stability under high-pressure and adiabatic conditions which leads to safer handling and performance reliability.^77^

However, one of the challenges in this area was associated with controlling the nitration process, which requires precise conditions to avoid excessive degradation of the cellulose structure. Further investigation into the nitration of CNCs, CNFs, and BNC could unlock their potential for high-performance applications, leveraging their inherent nanoscale properties and the added functionality of nitrate groups.

## 3. Environmental impact and recyclability of nanocellulose-based nitrated polymers in propellants

Nanocellulose-based nitrated polymers represent a sustainable alternative to conventional propellant materials with a significantly reduced environmental footprint. However, the limited biodegradability of nanocellulose-based nitrated polymers raises concerns over long-term ecological impacts and material accumulation in the environment. This was due to the nitration process, which involves the addition of nitric acid to cellulose, significantly alters its chemical structure.^39^ This makes nanocellulose-based nitrated polymers less prone to bacterial degradation compared to natural cellulose. Although nitrocellulose-based propellants produce toxic by-products, other commercial propellants generate even more harmful by-products compared to nitrocellulose. Compared to nitrocellulose-based propellants, nitramine-based propellants have a significantly higher capacity to produce hydrogen cyanide. For instance, an RDX-based propellant, incorporated into the M900 round for the 105 mm tank gun, produces significantly higher amounts of hydrogen cyanide and nitrous oxide.^78^

### 3.1. Nano-safety, toxicology, and bioaccumulation

Nanocellulose and its derivatives show great promise for applications like propellants, but their nanoscale nature brings important safety considerations particularly in terms of inhalation exposure, persistence, and biological interactions. Studies have shown that CNC can trigger inflammatory responses, particularly after chronic inhalation, with particle shape and size influencing immune reactions.^79^ Self-aggregation and bioaccumulation may further contribute to lung irritation upon excessive exposure. *In vitro* studies reveal that CNCs are non-cytotoxic at concentrations around 50 μg mL^−1^, but higher doses (>100 μg mL^−1^) can induce cell death and alter gene expression in mammalian cells.^80^

The toxicological properties of nanocellulose particles depend on numerous factors beyond dose and exposure including particle size, agglomeration, surface functionalisation, crystallinity, and charge.^81^ In one study, the cytotoxic effects of CNF were assessed using the MTT assay on keratinocyte (HaCaT) cells and fibroblast (HDF-α) across eight concentrations (39 to 5000 μg mL^−1^).^82^ Significant inhibition of HaCaT cell growth was observed at ≥156 μg mL^−1^, and HDF-α cells showed similar effects at ≥313 μg mL^−1^ compared to controls (*p* < 0.05). Despite this, no skin or eye irritation was detected in 3D tissue models.

While nanocellulose derivatives are generally considered low-risk, bioaccumulation remains a potential concern particularly *via* inhalation exposure.^83^ Although some studies suggest that nanocellulose has little acute toxicity and is likely non-hazardous when ingested in small quantities, additional chronic feeding studies are needed to assess long-term effects, especially on the gut microbiome and the absorption of essential micronutrients. For instance, CNF-50 and CNC-25 were associated with minimal or no cytotoxicity in a cellular model of intestinal epithelium, and no significant increase in reactive oxygen species production was observed at either a dose of CNF-50 or 0.75% w/w CNC-25.^84^ These primarily negative findings suggest that nanocellulose is likely non-toxic or only minimally toxic compared to other engineered nanomaterials that are generally regarded as safe.

### 3.2. Usage phase

Due to its nitrogen-rich molecular structure, nanocellulose-based nitrated polymers provides high energy release primarily through exothermic decomposition (heat of formation) rather than carbon oxidation—the dominant mechanism in traditional black powder.^85^ This combustion pathway generates fewer solid residues (*e.g.*, soot) and significantly reduced smoke compared to carbon-based propellants, resulting in clearer exhaust plumes and lower particulate emissions.^85^ The environmental advantages of nanocellulose-based polymer propellants can be further enhanced by eliminating perchlorate additives. Perchlorate (ClO_4_^−^) poses dual risks such as a competitive inhibitor of iodide uptake in the thyroid gland that can disrupt hormone synthesis leading to hypothyroidism and due to its high solubility and persistence, it readily contaminates groundwater through leaching from production sites or rainfall runoff, adversely affecting soil microbiomes and aquatic organisms.^85^ While this instability necessitates strict storage protocols for nitrocellulose, nanocellulose-based nitrated polymers alternatives maintain superior thermal stability without compromising combustion efficiency. By integrating these materials into propellant systems, the aerospace and defence industries can address stringent environmental and safety requirements, underscoring the broader sustainability benefits of nanocellulose-based alternatives (Table 3).

**Table 3:** Properties of nitrated CNFs and BNC for potential propellant application

Type of N–NC	Source	Condition	Properties	End-product	Ref.
N–BNC	*Nata de coco*	H_2_SO_4_ : HNO_3_ (3 : 1)	Nitrogen content of 12.64%	Powder	86
		35 °C	Rough & porous structure		
		22 min	Rate of decomposition ∼200 °C		
N–CNF	Cotton (40–100 mesh)	Mixture of AcOH (5.0 mL, 87.3 mmol), Ac_2_O (5.0 mL, 52.9 mmol), and 60% HNO_3_ (1.0 mL, 21.9 mmol or 2.5 mL, 54.8 mmol)	Nitrogen content of 12.64%	Freeze-dried solid	87
		0 °C	Surface area ∼260 m^2^ g^−1^		
		10 min	Nanofiber diameters ∼10–50 nm		
N–BNC	*Medusomyces gisevii* Sa-12	Mixed sulphuric–nitric acids (MA, 14% H_2_O content) & con. nitric acid in the presence of methylene chloride (NA + MC, 20 : 80)	Nitrogen content of 11.77–12.27%	Gel-film & Freeze-dried solid	88
		25–30 °C	Viscosity of 1086 mPa s		
		40 min	Heats of decomposition of 6.94–7.08 kJ g^−1^		
N–BNC	Biological nanocomposite at Southwest University of Science and Technology	Sol–gel method	Temperature decomposition of 224.8 °C	Powder crystal	4
		RDX, HMX, and CL-20 were dissolved into the above solutions in the mass ratio 1 : 1 to NBC	Heat release of 3346 J g^−1^		
		15 min			
N–CNF	Cotton-based cellulose powder (40–100 mesh)	H_2_O (8.6%), HNO_3_ (16.6%), H_2_SO_4_ (74.8%)	Nitrogen content of 9.0–13.7%	Powder	89
		10 min	Surface areas of 103.0 cm^2^ g^−1^		
		40 °C	The heat flow of 87.7 mW		
			*Q*_DSC_ values of 3.9 kJ g^−1^		
N–BNC	*Medusomyces gisevii* Sa-12	Acid to organic phase weight ratio – 1 : 50	Nitrogen content of 11.45%	Film	90
		80–90 °C	Viscosity of 1900 cP		
		60 min	As content of 0.10%		
N–BNC	*Acetobacter xylinum*	H_2_SO_4_, 98% (150 mL) and HNO_3_, 65% (100 mL)	Nitrogen content of 12.1%	Powder	91
		60 min	Web structure, ribbon-like fibre		
N–BNC	*Medusomyces gisevii* Sa-12	Ratio of BNC to mixed acid was 1 : 50	Nitrogen content of 10.96%	Powder	92
		25–30 °C	Viscosity of 916 cP		
		40 min			

#### 3.2.1. Combustion products and emissions

An important consideration in nanocellulose-based nitrated polymers application is the environmental impact of their combustion products and emissions. Nitrate esters undergo distinct decomposition pathways. The primary mechanism involves hydrolytic cleavage of the CO–NO_2_ bond (activation energy < 150 kJ mol^−1^), which liberates NO_2_ and generates alkoxy radicals while the secondary decomposition occurs *via* thermolysis at temperatures > 60 °C (activation energy > 150 kJ mol^−1^).^93^ Both pathways produce reactive NO_*x*_ species that, when stabilizer concentrations fall below critical thresholds (<20%), react with moisture to form acidic compounds. These acids initiate autocatalytic decomposition, ultimately leading to autoignition in nanocellulose-based nitrated polymer propellants.

The primary combustion products of cellulose-based nitrated polymers are typically carbon dioxide (CO_2_), nitrogen oxides (NO_*x*_), and water vapor.^94^ SO_2_ and NO_*x*_ are key precursors to acid rain and photochemical smog, whereas CO_2_, a major greenhouse gas (GHG), drives climate change by trapping heat in the atmosphere.^85^ In 2019, CO_2_ accounted for 75% of total global net anthropogenic greenhouse gas emissions, while nitrogen oxides (NO_*x*_) contributed approximately 4%.^95^ Unlike direct greenhouse gases, CO does not contribute to radiative forcing itself but indirectly enhances global warming by promoting the formation of other climate-warming gases.^85^ CO persists in the atmosphere for approximately three months (half-life), during which it undergoes oxidation to CO_2_ and facilitates tropospheric ozone (O_3_) production, collectively altering atmospheric chemical processes.^96^

These emissions are common to the combustion of organic materials and are generally considered to be less harmful than those from fossil fuel-based propellants, as cellulose is a carbon-neutral material.^94^ However, the nitration process introduces additional complexities. The presence of nitrogen in the polymer matrix results in the release of nitrogen-based compounds such as nitrous oxide (N_2_O) and nitric oxide (NO), both of which are potent pollutants.^97^ Nitrous oxide, in particular, is a greenhouse gas with a significantly higher global warming potential compared to CO_2_. Additionally, the production of these combustion byproducts can vary depending on the specific formulation of the nanocellulose-based propellant and the combustion conditions, including temperature and oxygen availability.

To enhance the sustainability of nanocellulose-based nitrated polymers in propellants, ongoing research is focused on improving the combustion characteristics and minimizing harmful emissions. This includes the development of advanced additives that can promote cleaner combustion and the use of combustion chambers designed to reduce the formation of toxic byproducts. By addressing the environmental impact of combustion products and emissions, nanocellulose-based propellants may serve as a viable, more sustainable alternative to traditional materials, contributing to a greener future in propulsion technologies.

#### 3.2.2. Residue and particulate matter

The environmental impact of their combustion or decomposition in propulsion systems also includes the formation of residues and particulate matter, which can have significant implications for both air quality and ecosystem health.^98^ The traditional black powder used in propellants generated smoke, which led to incomplete combustion of a significant portion of the propellant, wasting up to 57% of its potential energy and reducing the muzzle velocity for a given charge weight.^3^ The residue from the powder often ignited, causing the destruction of the weapon and sometimes resulting in injury or death to the person firing it. During combustion, black powder undergoes a chemical reaction that releases several gases, contributing to the dangerous buildup of pressure within the firearm.

When these nanocellulose-based nitrated polymers are used as propellant materials, their combustion often results in the formation of solid residues, which are composed of various inorganic and organic byproducts. The nature and quantity of these residues depend on the specific formulation and the conditions under which the combustion occurs.^99^ When nitro-nanocellulose is subjected to external energy, it will thermally decompose at a specific heating rate, and any changes in the external energy will affect the heating rate. Nanocellulose-based nitrated polymers have a clear limit on the maximum stacking volume during transportation and storage. This limitation exists because changes in the sample volume within a sealed environment can significantly impact their thermal decomposition.^39^ If the storage capacity is exceeded, excessive heat will increase its heat dissipation. However, when heat dissipation is insufficient, heat accumulation occurs, reducing their thermal safety.

The incomplete combustion of particulates, often in the form of fine soot or ash, can be harmful to both the environment and human health, especially when they are emitted in large quantities.^100^ Fine particulate matter (PM2.5) is known to cause respiratory problems and has been associated with cardiovascular diseases when inhaled.^101^ Under normal temperature and pressure, nitro-nanocellulose is stable. However, it is shock-sensitive and can break down when heated, releasing poisonous gases such as nitrogen oxides and carbon monoxide.^39^ Both dry and damp nitrocellulose easily reacts with strong oxidizing agents, including strong acids and bases, which highlights the need for stabilizers. The particulate matter generated during the combustion of cellulose-based nitrated polymers may also include toxic or carcinogenic compounds. For example, the formation of nitroaromatic compounds, which can occur during the thermal decomposition of the nitrated cellulose structure, can increase the toxicity of the residues.^102^ These substances are particularly concerning as they can persist in the environment and may bioaccumulate in plants and animals, leading to long-term ecological and health impacts. Furthermore, the size and composition of the particulate matter can vary, with some particles being small enough to penetrate deep into the lungs, contributing to air pollution and human health risks in areas where propellants are frequently used, such as in military or aerospace activities.

Mitigating the environmental impact of residue and particulate matter from nanocellulose-based nitrated polymers requires careful optimization of their combustion and decomposition processes. Research efforts are focused on enhancing the combustion efficiency of these materials to ensure complete combustion and minimize the generation of harmful residues. Additionally, the development of novel additives or treatments to reduce the formation of toxic byproducts during combustion could significantly improve the sustainability of cellulose-based propellants. By addressing the challenge of residue and particulate emissions, these materials could become a more viable, environmentally friendly alternative to traditional fossil-fuel-based propellants, supporting both cleaner propulsion technologies and sustainable industrial practices.

### 3.3. Disposal and end-of-life considerations

The disposal and end-of-life management of CNFs, CNCs, and BNC, is a critical consideration in evaluating their environmental impact and sustainability in propellant applications. The nitration process, which imparts explosive properties to these cellulose derivatives, can alter their degradation pathways and create unique challenges in their disposal and environmental impact at the end of their life cycle.^30^

One of the primary environmental advantages of nanocellulose-based materials is their natural biodegradability. Under optimal conditions, cellulose and its derivatives are capable of breaking down in natural environments through microbial action, which results in the release of non-toxic byproducts such as carbon dioxide and water.^10^ This makes nanocellulose-based nitrated polymers an attractive alternative to petroleum-based polymers, which can persist in the environment for extended periods, contributing to long-term pollution. However, the nitration process introduces nitrogen-containing functional groups to the nanocellulose structure,^41^ which can complicate biodegradation. Nitrogen-rich residues, such as nanocellulose-based nitrated polymers, may persist longer in the environment, potentially affecting soil and water quality. Thus, while nanocellulose-based nitrated polymers can degrade over time, the rate and extent of this process need to be carefully evaluated, especially in specific environments where the presence of nitrogen-based byproducts could lead to localized contamination.

While nanocellulose itself is highly recyclable, the presence of the nitrated groups complicates traditional recycling processes. However, recent advances in green chemistry and material science have suggested that it may be possible to recover and recycle some forms of nitro-nanocellulose. For instance, the use of recycled egg carton pulp as a sustainable material for briquette production, enhanced with nitrocellulose as an accelerant was conducted.^103^ The addition of nitrocellulose, known for its fast ignition properties, accelerates combustion, improving the efficiency of the briquettes. By combining recyclable egg carton pulp with nitrocellulose, this method not only enhances the energy production process but also emphasizes the importance of recycling in reducing the environmental impact, making the entire process more eco-friendly and sustainable.

Ultimately, addressing the end-of-life considerations for cellulose-based nitrated polymers requires a comprehensive understanding of their degradation behavior, toxicity of byproducts, and potential for recycling. By developing more efficient disposal methods, optimizing biodegradation processes, and exploring innovative recycling techniques, the environmental impact of these materials at the end of their life cycle can be minimized. These efforts will contribute to the broader goal of promoting the sustainability of nanocellulose-based propellants as a viable alternative to conventional, less environmentally friendly materials.

#### 3.3.1. Degradation in the environment

The degradation reaction of nitro-nanocellulose begins with the scission of the O–NO_2_ bond, releasing NO_2_, which immediately complexes to form a nitrato group (CONO_2_).^39^ This group rapidly participates in a reaction that produces oxidation products. The presence of water in the surrounding environment or within the nitro-nanocellulose promotes a hydrolysis reaction of the O–NO_2_ bond, resulting in the formation of nitric acid (HNO_3_). This acid accelerates the reaction through catalytic action, releasing more NO_2_ and leading to a cyclical pathway that can result in uncontrolled self-heating consequences.

In addition, presence of fixed acid and water content in nitro-nanocellulose-based nitrated polymers propellants can make them prone to autocatalytic degradation over time, posing a significant stability concern.^3^ To address this, stabilizers are added during manufacturing to extend the shelf-life and improve the stability of the propellants. These stabilizers neutralize nitrous gases released during decomposition, which can otherwise accelerate degradation. By trapping these gases, stabilizers prevent further breakdown, helping maintain the propellants' quality and safety for use in ammunition and pyrotechnics.^3^

Nitrocellulose is usually shipped wetted with water or alcohol and is soluble in acetone but insoluble in water.^104^ It is also more soluble in ammonia than in aqueous alkaline solutions. Separating nitro-nanocellulose fine particles from wastewater is challenging due to the wide variation in particle sizes, ranging from sub-micron to a few millimeters. Additionally, the wastewater is corrosive and hot, making the separation process even more difficult. Current nitrocellulose waste has been shown to be remarkably stable, and stringent military specifications further complicate its reuse.^104^ For instance, in aquatic environments, the solubility and dispersion of nanocellulose-based nitrated polymers could affect their biodegradation. The persistence of these nanocellulose-based nitrated polymers in water could lead to localized contamination, particularly if the nitrogen-based byproducts, such as nitrous oxide or nitric acid, are released into the ecosystem.

However, a few methods have been developed to overcome this issue such as photodecomposition laser, chemical precipitation, alkaline and acid decomposition, and biological treatment. The treatment process focused on efficiently recovering nitrocellulose using a dissolved air flotation (DAF) unit, with different operating conditions tested for various effluent characteristics, both with and without flocculating agents.^104^ Recovery reached 80% by flotation without chemicals and 87% using a cationic polymer. These results provide a solid foundation for the full-scale industrial treatment unit. The pilot DAF system separated up to 80% of nitrocellulose from the washing section effluent and 55% from the end-pipe effluent, increasing to 87% with the addition of cationic polymer. The process could recover up to 50 tons per year of pure nitrocellulose based on the actual production capacity. These findings highlight an effective treatment method for recovering nitrocellulose from industrial effluents.

In another study, the simultaneous oxidative degradation of toxic acid wastewater generated during nitrocellulose production, using Mn^2+^ released from low-grade MnO_2_ ore as an oxidant was conducted.^105^ The study highlights the environmental challenge posed by the toxic wastewater, which contains nitrocellulose byproducts, including nitrogen-based compounds. By utilizing Mn^2+^ as an oxidant, the process effectively degrades the contaminants, including nitrocellulose-related compounds, while reducing the environmental impact. The use of low-grade MnO_2_ ore as a cost-effective and sustainable oxidant is emphasized as a promising solution for treating such hazardous wastewater.

The interaction between nanocellulose-based nitrated polymers and environmental conditions like sunlight (UV radiation) can also play a significant role in their degradation. Photodegradation processes, where the polymer material is broken down by UV radiation.^106^ The UV light degradation of cellulose nitrate films was investigated under conditions of artificially accelerated photooxidation, alongside the degradation of ethylcellulose to eliminate the reactivity of nitro groups.^107^ Infrared spectroscopy analyses revealed chemical modifications caused by the photooxidation of cellulose nitrate films, including de-nitration and the formation of oxidation photoproducts, such as lactones and anhydrides. The study highlights the chemical changes that occur during the photooxidation of cellulose nitrate, leading to the formation of gluconolactone and anhydrides as key oxidation products. However, UV radiation may not be as effective in degrading the nitrated groups, resulting in incomplete degradation and the potential for toxic byproducts to persist in the environment.

In order to enhance the sustainability of nanocellulose-based nitrated polymers in propellants, strategies for accelerating and controlling their environmental degradation are essential. This could involve optimizing the chemical structure to make the nitrated groups more amenable to microbial degradation or incorporating additives that can enhance the breakdown process. Additionally, research into the long-term environmental fate of these materials, including the potential for bioaccumulation and ecological toxicity, is crucial to ensure that their environmental footprint is minimized. As the use of nanocellulose-based nitrated polymers grows in the field of propulsion, it will be essential to develop best practices for managing their degradation to ensure minimal impact on ecosystems and human health.

#### 3.3.2. Potential for recycling or reuse

Nanocellulose-based nitrated polymers offer several advantages in terms of biodegradability and renewable sourcing, the presence of nitrated groups in these polymers introduces additional challenges to traditional recycling and reuse processes. Understanding the feasibility and methods for recycling these materials is essential for promoting a circular economy and reducing the environmental footprint of cellulose-based propellants.

Conventional recycling methods that work for non-nitrated nanocellulose may not be directly applicable to nanocellulose-based nitrated polymers due to the altered chemical and physical properties of the nitrated forms. As a result, new approaches to recycling these materials must be developed, taking into account the challenges posed by their modified chemical structure. One potential pathway for recycling nanocellulose-based nitrated polymers could involve the recovery of the cellulose matrix after the removal of the nitrated functional groups. This process could involve chemical treatment or enzymatic hydrolysis,^108^ which could break down the nitrated cellulose into non-explosive components, enabling the reuse of the cellulose fraction in other applications.

Specific microorganisms are capable of degrading nitrated nanocellulose compounds under both anaerobic and aerobic conditions. For instance, the most promising microorganisms for efficient nitrocellulose degradation include bacteria from the *Desulfovibrio* genus, fungi such as *Fusarium solani* and *Sclerotium rolfsii*, as well as their co-cultivation.^109^ Biotransformed nitrocellulose under anaerobic conditions showed nitrogen content similar to non-explosive nitrocellulose, suggesting that anaerobic treatment could be a feasible process for neutralizing NC. In addition, effluents from nanocellulose-based nitrated production contain significant amounts of sulfates, making sulfate-reducing bacteria (SRB) potential agents for their decomposition. Furthermore, factors including the composition of the medium, culture conditions, accumulation of inhibitory products affecting key enzymes, and the presence of functional groups in sterically inaccessible regions influencing the limited degradation of nanocellulose-based nitrated polymers.

The development of effective recycling or reuse strategies for nanocellulose-based nitrated polymers requires addressing several technical and environmental challenges. Chief among these is the need to ensure that the recycling process does not inadvertently lead to the release of toxic or hazardous byproducts, particularly those related to the decomposition of the nitrated groups. Moreover, the economic viability of recycling or repurposing these materials must be considered, as the cost of processing may outweigh the environmental benefits if not optimized. Ultimately, enhancing the recyclability of these materials will play a key role in reducing their environmental impact and ensuring that they contribute to a circular economy.

## 4. Sustainability and advantages of nanocellulose-based nitrated polymers in propellants

### 4.1. Resource efficiency

The use of nanocellulose-based nitrated polymers, such as those derived from CNFs, CNCs, and BNC, in propellants exemplifies a significant advancement in resource efficiency. These materials originate from renewable resources, including agricultural waste, wood pulp, and microbial fermentation, which reduces dependency on non-renewable, petroleum-based feedstocks.^110^ The ability to harness widely available and renewable biomass sources ensures a steady supply chain with minimal environmental disruption, making these nanocellulose-based materials a sustainable alternative for high-energy applications. Furthermore, their production processes can be tailored to use industrial byproducts or low-value agricultural residues, turning waste into a valuable resource.

The extraction and production methods for CNFs, CNCs, and BNC offer additional resource-efficient benefits. CNFs and CNCs have been successfully isolated from diverse natural sources including wood, cotton, hemp, flax, wheat straw, sugar beet, potato tuber, mulberry bark, ramie, algae, and tunicin.^110^ The production process involves the transformation of native cellulose, which typically exists at the centimeter scale, into nanoscale structures (ranging from nanometers to micrometers). Among various extraction methods, acid hydrolysis has emerged as the most widely adopted chemical treatment for cellulose restructuring and nanomaterial isolation. This process effectively breaks down the hierarchical structure of cellulose, removing amorphous regions while preserving the crystalline domains to yield high-purity nanocellulose materials.

Similarly, BNC is synthesized various bacterial species, including those belonging to the genera *Komagataeibacter*, *Acetobacter*, *Agrobacterium*, *Pseudomonas*, *Sarcina*, and others.^111^ Cultivating specific bacterial strains, such as *Komagataeibacter xylinus*, *Gluconacetobacter hansenii*, and *Gluconacetobacter xylinus*, in an appropriate culture medium under controlled conditions is essential for achieving high BNC yields with the desired characteristics.^112^ BNC production can even be adapted to utilize nutrient-rich waste streams, such as agricultural runoff or industrial effluents, reducing the demand for virgin raw materials and enhancing the circularity of the production process.

#### 4.1.1. Renewable *vs.* non-renewable raw materials

One of the most significant sustainability advantages of nanocellulose-based nitrated polymers, such as those derived from CNFs, CNCs, and BC, is their reliance on renewable raw materials. These polymers are synthesized from cellulose, the most abundant natural polymer on Earth, sourced from plants or microbial fermentation.^113^ This stands in stark contrast to conventional propellants, which often rely on synthetic polymers derived from finite fossil resources. By utilizing nanocellulose from renewable sources, the production of these nitrated-nanocellulose polymers reduces dependence on non-renewable materials, helping to conserve critical fossil reserves and decrease the carbon footprint associated with their extraction and processing.

The renewable origins of CNFs, CNCs, and BNC also offer a sustainable alternative for addressing the environmental challenges posed by synthetic energetic materials. Unlike petroleum-based polymers, which require energy-intensive processing and contribute significantly to greenhouse gas emissions, cellulose-based materials are part of a natural carbon cycle.^114^ CNFs and CNCs can be sustainably derived from various biowaste sources, including tea leaf waste fibers, grass waste, and sugarcane straw.^110^ In addition, green algae particularly promising alternative sources for nanocellulose extraction. As photosynthetic microorganisms ubiquitously distributed in both freshwater and marine ecosystems, green algae exhibit remarkable morphological diversity and ecological adaptability. This enhanced crystallinity, attributed to the unique cellulose biosynthesis pathways in algae, results in superior mechanical properties and thermal stability, making algal-derived nanocellulose particularly attractive for high-performance applications.

BNC is particularly notable in this regard, as it is produced through microbial fermentation, bypassing the need for plant cultivation altogether. This method not only eliminates the land-use challenges associated with traditional cellulose sources but also allows BNC production to utilize industrial byproducts or waste streams, such as food processing residues or agricultural runoff.^112^ Research has predominantly focused on natural carbon sources derived from fruit wastes and crop residues as sustainable and cost-effective alternatives to the conventional Hestrin–Schramm (HS) medium, which relies on expensive components such as glucose, yeast extract, and peptone.^115^ Notable examples include citrus juices from oranges (5.9 g L^−1^) and grapefruits (6.7 g L^−1^), date fruit waste extracts (3.9 g L^−1^), pineapple waste (2.0 g L^−1^), rotten bananas (5.34 g L^−1^), and mango waste (0.52 g L^−1^). Additionally, crop residues such as potato peel waste (2.6 g L^−1^), coconut water (3.9 g L^−1^), and molasses have demonstrated promising results.^115^ These substrates are particularly advantageous due to their high concentrations of fermentable sugars, nitrogen, and essential trace elements, which are critical for enhancing BNC productivity on an industrial scale.

This closed-loop approach minimizes waste while providing a renewable feedstock for nitrated polymers. Similarly, CNFs, CNCs, and BNC derived from agricultural waste or sustainably managed forests contribute to resource efficiency by repurposing materials that might otherwise go unused or be discarded. In contrast, non-renewable raw materials commonly used in traditional propellants are associated with significant environmental drawbacks, including habitat destruction, resource depletion, and high carbon emissions. The shift toward cellulose-based nitrated polymers leverages renewable resources while aligning with global efforts to develop greener, more sustainable materials. By replacing non-renewable inputs with cellulose-based alternatives, the propellant industry can make meaningful progress toward reducing its environmental footprint, supporting a transition to a more sustainable and circular economy. This transition not only ensures the availability of essential materials for high-performance applications but also contributes to long-term environmental stewardship.

#### 4.1.2. Water, acids, and energy consumption in production

Traditional processes for producing nanocellulose-based nitrated polymers often involve high energy input and the use of concentrated acids, resulting in significant waste and environmental impact. However, advances in green chemistry and process optimization have made it possible to reduce these demands, aligning the production of cellulose-based materials with sustainable manufacturing practices.

Water consumption, a critical factor in sustainable production, has been minimized in modern cellulose processing techniques. For instance, the extraction of CNFs and CNCs has been adapted to recycle process water, significantly reducing overall water usage.^116^ Furthermore, BNC production in bioreactors requires relatively low water input compared to traditional plant-based cellulose sources, as it avoids the large-scale irrigation and agricultural practices necessary for crops like cotton.^117^ Additionally, BNC production can utilize nutrient-rich wastewater or byproducts from other industries, further decreasing water demand and promoting circular resource use.

The use of acids in the production process, particularly in the hydrolysis of cellulose to create CNCs and during nitration to produce nitro-cellulose nanocrystals (NCNC), has also been a focus of sustainability improvements. Various acids, including HCl, H_2_SO_4_, HBr, and H_3_PO_4_, have been used; however, H_2_SO_4_ is generally utilized because of its ability to isolate CNC and facilitate the dispersion of nanocellulose into a stable colloidal system by the esterification of hydroxyl groups by sulfate ions.^53^ Acid hydrolysis can generate significant chemical waste. However, recent developments in acid recovery and reuse systems have greatly reduced the environmental impact of this step, enabling the efficient recycling of acids without compromising product quality. Similarly, for nitration, optimized acid mixtures and reaction conditions have been developed to lower acid concentrations and minimize waste, contributing to greener production practices.^87^

Energy consumption during the production of CNFs, CNCs, and BNC has been addressed through the adoption of energy-efficient technologies. For CNFs, mechanical fibrillation processes traditionally consumed large amounts of energy, but advancements such as enzymatic pretreatment or chemical-assisted processing have significantly lowered energy requirements.^118^ In CNC production, process optimizations like reduced reaction times and improved acid efficiency have minimized energy input without sacrificing yield. BNC production is inherently energy-efficient due to its biogenic nature, with controlled fermentation processes that can be powered by renewable energy sources to further reduce the carbon footprint.

By addressing water, acids, and energy consumption, the production of nanocellulose-based nitrated polymers for propellants achieves a balance between performance and environmental responsibility. These advancements ensure that CNF, CNC, and BNC not only offer high energy output and tunable properties but also align with global sustainability goals, making them an ideal choice for environmentally conscious energetic materials. Through continued innovation, the propellant industry can further reduce its reliance on finite resources and harmful processes, contributing to a cleaner and more sustainable future.

### 4.2. Life cycle assessment

**Fig. 4:**
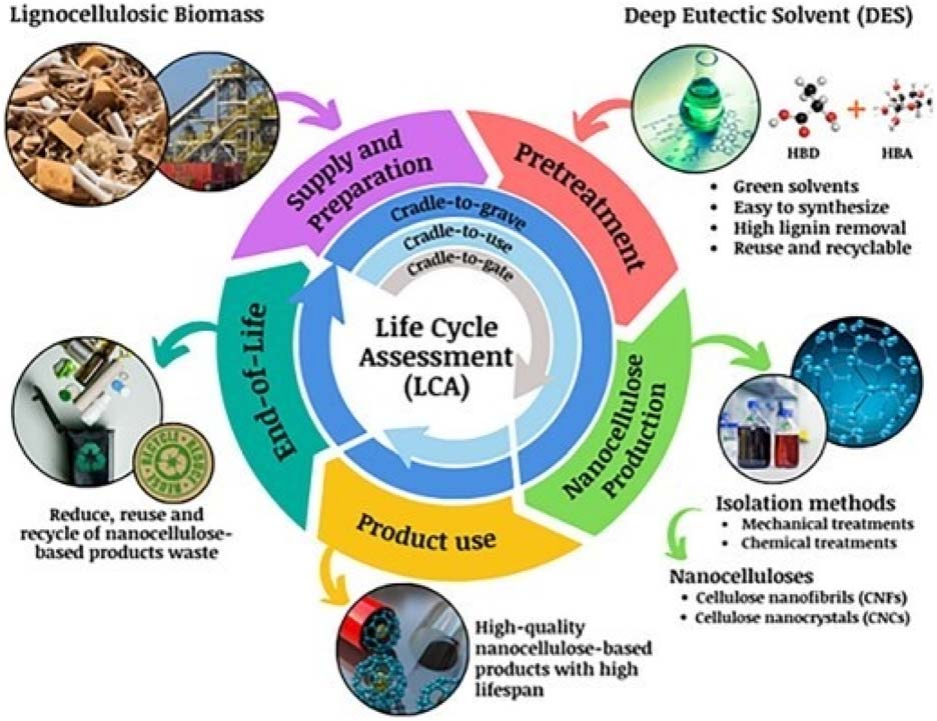
Life cycle assessment (LCA) of CNCs, CNFs, and BNC. Reproduced from ref. 119 with permission from the Taylor & Francis.

Life cycle assessment (LCA) is a critical tool for evaluating the environmental impact of nanocellulose-based nitrated polymers, such as those derived from CNFs, CNCs, and BNCs, across their entire lifecycle from raw material sourcing to end-of-life degradation (Fig. 4). Incorporating LCA into the development and deployment of these materials provides insights into their sustainability advantages compared to traditional synthetic polymers used in propellants. Key considerations include resource extraction, production processes, use-phase performance, and final disposal, all of which contribute to the overall environmental footprint.

A significant advantage highlighted by LCA is the renewable and sustainable nature of cellulose feedstocks. Traditional propellants are typically based on fossil fuel derived polymers and ingredients, which contribute significantly to greenhouse gas emissions during both production and combustion.^120^ To mitigate environmental impacts, decarbonization strategies were pursued by substituting fossil-based feedstocks with renewable nanocellulose.

CNCs, CNFs, and BNC demonstrate significantly lower environmental impacts during the raw material acquisition phase compared to fossil fuel-based alternatives. This offers a lower-carbon alternative that can substantially reduce the carbon footprint of propellant formulations.^110^ In addition, adopting renewable feedstocks aligns with circular economy principles, minimizing waste, promoting resource reuse, and decreasing reliance on virgin materials, thus supporting a more sustainable life cycle for propellant systems.^121^ LCA studies consistently show reduced greenhouse gas emissions and energy requirements when renewable nanocellulose sources are used in place of non-renewable feedstocks.

The environmental impact of civilian emulsion explosives (EEs) formulated with nitrocellulose derived from recycled ammunition, benchmarking the results against traditional EEs and those containing standard nitrocellulose. LCA was performed using the CML Baseline v3.07 methodology and the Ecoinvent 3.8 database, with inventory data sourced from Polish operations.^122^ They found that using virgin NC increased the overall environmental impact of EEs by about 4.5%, whereas substituting recycled NC reduced it by approximately 4.99%.^122^

A cradle-to-gate LCA conducted by comparing three production routes for cellulose nanofibrils (CNF) derived from wood pulp: enzymatic pretreatment with microfluidization, carboxymethylation pretreatment with microfluidization, and a no-pretreatment route using homogenization.^123^ From the results, carboxymethylation route exhibited the highest environmental impacts, primarily due to heavy solvent use from crude oil sources. In contrast, the enzymatic and no-pretreatment routes demonstrated significantly lower impacts of similar magnitude. Sensitivity analysis revealed that the no-pretreatment route's performance was highly sensitive to the electricity mix, while the carboxymethylation route was most affected by solvent recovery efficiency.

Quantitatively, Ankerfors *et al.* estimated electricity consumption for enzymatic and carboxymethylation pretreatment plus microfluidization at approximately 8.0–8.4 MJ per kg CNF, which was used as the baseline in the sensitivity scenarios.^124^ Meanwhile, the no-pretreatment homologation route consumed around 97 MJ per kg CNF, though reported values varied between 72–108 MJ kg^−1^; the lower end (72 MJ kg^−1^) was adopted as a baseline for efficient future production.^123^ These findings illustrate how selecting low-impact pretreatments and optimizing key process variables such as energy sourcing and solvent recovery can dramatically reduce the environmental footprint. They also provide a sound foundation for extending LCA modeling to nitrated nanocellulose propellants using similarly optimized, green process pathways.

LCA of CNFs produced from bleached hardwood kraft pulp *via* three different pre-treatments before mechanical homogenization fully mechanical (valley beating + homogenization), enzymatic pretreatment (with endoglucanases at ∼50 °C), and TEMPO-mediated oxidation prior to homogenization were conducted.^125^ Furthermore, the conclusions remain consistent whether using 1 kg of CNF as the functional unit or adjusting based on the cellulose amount required for paper-strengthening functions. However, it is important to note that all modelled scenarios assume laboratory-scale, well-established conditions, which may differ significantly from optimized industrial operations.

By synergistically combining CNFs, CNCs, and BNC, it improves tensile strength, reduces shrinkage, and mitigates cracking while lowering the carbon footprint of cement production.^126^ LCA confirm its environmental benefits, including reduced energy use and greenhouse gas emissions. Despite its promise, challenges such as moisture sensitivity, high energy demands for extraction, and scalability limitations must be addressed to facilitate widespread adoption. As a novel, eco-friendly material, combo nanocellulose aligns with sustainable construction goals by enhancing recyclability, reducing lifetime costs, and minimizing environmental impact. Its versatility spans structural components to insulating coatings, though future research must prioritize scalable production, regulatory standardization, and industrial integration to fully realize its potential in green infrastructure. This innovation paves the way for resilient, low-carbon construction while meeting stringent sustainability targets. In summary, life cycle assessments of CNFs, CNCs, and BNC highlight their environmental superiority over traditional synthetic polymers used in propellants. Continued refinement of LCA methodologies and expanded datasets will help quantify these advantages more precisely, guiding the development of greener propellant technologies.

#### 4.2.1. Regulatory framework

Under Regulation (EC) 1907/2006, any manufactured or imported substance including nanocellulose requiring tonnage ≥ 1 t per year must be registered with ECHA.^127^ The free movement of goods, persons, services, and capital underpins the EU's internal market. As chemicals fall under the category of “goods,” their free movement is naturally included within this framework.

Nanotechnologies especially nanocellulose are gaining prominence, prompting growing regulatory attention. Although the EU has no specific legislation for nanocellulose, general chemical regulations apply. The cornerstone of these regulations is Regulation (EC) no. 1907/2006 (REACH), which governs the registration, evaluation, authorisation, and restriction of chemicals. The European Commission (2012) defines nanomaterials under REACH as materials whether natural, incidental, or manufactured that contain particles in an unbound state, or as aggregates or agglomerates, where 50% or more of the particles by number size distribution have at least one dimension between 1 and 100 nm.^128^ In particular cases driven by environmental, health, safety, or competitiveness concerns, this threshold may be adjusted to between 1% and 50%.

Despite this comprehensive framework, there remains no EU legislation tailored specifically to nanocellulose meaning that REACH principles must be applied broadly to these materials.

#### 4.2.2. Performance *vs.* environmental trade-offs

The adoption of nanocellulose-based nitrated polymers, such as CNFs, CNCs, and BNC, in propellants represents a promising step toward environmentally sustainable energetic materials. However, the integration of these materials involves inherent trade-offs between achieving high-performance energy outputs and minimizing environmental impacts. Understanding these trade-offs is essential for optimizing their applications in aerospace, defence, and industrial propulsion systems while adhering to sustainability goals.

Emerging metrics in propellant research focus on enhancing performance, minimizing environmental impact, and enabling new capabilities. Key indicators include burn rate, pressure exponent, combustion efficiency, and eco-friendliness.^129^ The key areas of research for nanocellulose-based nitrated polymer propellants include the crystallinity index, particle size, surface modification, and dispersibility.^130^ These metrics are crucial for maximizing the energetic properties of nanocellulose-based propellants and ensuring their suitability for various applications. The crystallinity index reflects the proportion of crystalline regions within the nanocellulose structure, and higher values generally lead to improved mechanical properties and enhanced energy release in energetic materials.^131^ Furthermore, the particle size and shape critically influence surface area and reactivity of nanocellulose-based nitrated polymer.^132^ For instance, the BET analysis revealed that the specific surface areas of NC, CNF, and NCNF were 9.8, 107.2, and 103.0 m^2^ g^−1^, respectively showing that CNF and NCNF have roughly ten times the surface area of NC.^89^ Notably, the burning speed of NCNF was 3.5 times higher than that of NC, demonstrating how higher surface area directly contributes to combustion enhancement.

From a performance perspective, nanocellulose-based nitrated polymers offer tunable properties that allow them to meet or exceed the energy outputs of traditional synthetic energetic materials. The high crystallinity of CNCs and BNC, as well as the tailored fibrillar structure of CNFs, contribute to efficient nitration, resulting in nitrated nanocellulose polymers with excellent energy density and combustion characteristics.^133^ Furthermore, these materials exhibit superior mechanical properties and thermal stability, which enhance their suitability for high-performance propellant formulations. However, achieving these desirable traits often requires precise control over production parameters, such as reaction conditions during nitration, which can increase resource consumption or generate additional chemical waste.

Another critical trade-off involves the scalability of production processes. While BNC offers distinct environmental advantages due to its fermentation-based synthesis, scaling up BNC production to meet industrial demands for propellants remains a challenge, particularly in terms of cost and infrastructure requirements.^134^ Similarly, the production of highly crystalline CNCs involves acid hydrolysis processes that, while efficient at laboratory scales, may result in environmental burdens when scaled up without appropriate waste management systems.^135^ These challenges highlight the need for continued innovation to optimize production methods while maintaining the balance between performance and environmental impact.

Ultimately, the trade-offs between performance and environmental impact underscore the importance of a holistic approach to material development and application. By leveraging LCA and integrating green chemistry principles into production processes, it is possible to maximize the benefits of cellulose-based nitrated polymers while minimizing their environmental drawbacks. Research efforts aimed at improving production efficiency, reducing resource consumption, and enhancing the scalability of CNFs, CNCs, and BNC will be critical in ensuring these materials achieve their full potential as sustainable alternatives in the propellant industry.

### 4.3. Comparison with nanocellulose-based nitrated polymers traditional propellant materials

#### 4.3.1. Resource

Nanocellulose-based nitrated polymers derived from CNF, CNC, and BNC offer a compelling alternative to traditional propellant materials, particularly in the context of sustainability, environmental impact, and performance. Traditional propellant materials, typically composed of synthetic polymers like nitrocellulose from fossil fuels or thermoplastic binders, are highly effective in terms of energy output but come with significant drawbacks, including a reliance on non-renewable resources, long-term environmental persistence, and complex waste management challenges.^136^ The adoption of nanocellulose-based alternatives represents an opportunity to address these issues without compromising performance.

A primary distinction between nanocellulose-based and traditional propellant materials lies in their feedstock sources. CNFs, CNCs, and BNC are derived from renewable and abundant sources such as plant biomass and microbial fermentation, making them inherently more sustainable. In contrast, traditional materials rely heavily on petroleum-derived inputs, which are finite and associated with high carbon emissions during extraction and processing.

Traditional solid rocket propellants have long relied on petroleum-based formulations, with ammonium perchlorate (AP)-based composite propellants serving as the industry workhorse for over 50 years. These propellants typically consist of multi-modal AP grains (20–200 μm) embedded in a hydroxyl-terminated polybutadiene (HTPB) matrix, which remains the most widely understood fuel in modern hybrid rockets.^15^ While alternative propellants like hydroxylammonium nitrate (HAN) offer advantages such as lower handling hazards and higher energy density, they present challenges in atomization due to their viscous nature, particularly for micro-thruster applications. Other petroleum derivatives like hydrazine (an endothermic compound with high calorific value) and methane (showing 10 s higher specific impulse than kerosene) have been explored, along with paraffin blends that demonstrate 3–4 times greater regression rates than HTPB while producing cleaner exhaust gases.^137^ However, these traditional petroleum-based propellants face significant disadvantages compared to nitro-nanocellulose alternatives, particularly in terms of environmental impact, as they rely on non-renewable resources and often produce more hazardous combustion byproducts than nanocellulose-derived formulations.

#### 4.3.2. Energy performance

In terms of energy performance, nanocellulose-based nitrated polymers offer competitive characteristics. The high surface area, crystallinity, and tunable structure of CNFs and CNCs^138^ allow for efficient nitration, resulting in high energy densities and combustion efficiency. BNC with its highly ordered nanoscale network, provides excellent mechanical properties and a consistent structure for nitration, contributing to its potential as a high-performance material.^111^ While traditional materials may still achieve higher peak energy outputs in some applications, nanocellulose-based polymers offer a promising balance of performance and sustainability, making them suitable for modern propellant systems where environmental considerations are a priority.

Manufacturing processes also highlight key differences between these material types. Traditional propellant materials often involve energy-intensive and hazardous chemical processes, such as the production of nitrocellulose or synthetic binders, which generate significant waste and emissions. In contrast, advancements in green chemistry have enabled more sustainable production pathways for CNFs, CNCs, and BNC.^135^ For example, acid recovery systems and enzymatic treatments in nanocellulose processing reduce the environmental burden while maintaining high yields. However, challenges remain in scaling up these processes to industrial levels, particularly for BNC, which requires controlled fermentation systems, and CNC, which involves acid hydrolysis techniques that must be carefully managed to minimize waste.

Overall, nanocellulose-based nitrated polymers represent a significant advancement over traditional materials in terms of environmental sustainability, with comparable performance for many applications. By addressing challenges related to scalability, production efficiency, and waste management, CNF, CNC, and BNC can serve as viable alternatives, aligning with global efforts to transition toward greener technologies. As the demand for sustainable high-performance materials grows, cellulose-based polymers are poised to play a crucial role in redefining the future of propellant technologies.

### 4.4. Green chemistry principles in synthesis

The growing global demand for sustainable materials has driven increased interest in cellulose nanomaterials (CNMs) as eco-friendly alternatives to conventional petroleum-based products. Green synthesis methods are particularly promising for nanocellulose production, with enzymatic pretreatment emerging as a safe and effective alternative to harsh chemical processes. Key to this approach is enzyme synergy, where the LPMO AA9 auxiliary enzyme demonstrates remarkable oxidative capability in cleaving β-1,4-glycosidic bonds and modifying fiber structures. Recent advances combine cellulases and xylanases with LPMO AA9 to enhance pretreatment efficiency for cellulose nanocrystal production.^139^

Cellulases specifically catalyze the hydrolysis of cellulose, selectively depolymerizing the amorphous regions up to 30 times faster than crystalline regions.^140^ These enzymes are typically sourced from cellulolytic organisms such as *Clostridium*, *Trichoderma*, and *Aspergillus*. Different process configurations and the choice between producing CNF or CNC depend on the cellulose source and desired material, using diverse enzyme mixes: for CNF, combinations include xylanase, cellulase, *Xanthomonas axonopodis* lysate, and pectinase, whereas CNC production often employs *Aspergillus niger* cellulase or similar strains.^141^

For example, the synergistic use of cellulase with xylanase accelerates hydrolysis and enhances efficiency compared to cellulase alone, although excessive xylanase does not further boost yield.^142^ Moreover, the cellulase–xylanase ratio significantly influences CNC morphology: a surplus of cellulase yields spherical CNCs (∼40–70 nm diameter), while higher xylanase levels generate rod-like morphologies with lengths of 750–1000 nm.^143^ These findings confirm that process tuning including enzyme type, concentration, and hydrolysis time enables precise control over nanocellulose dimensions and shape.

Life-cycle assessments reinforce the environmental advantages of enzymatic pretreatment. Piccinno *et al.*^144^ found that producing microfibrillated cellulose (MFC) from carrot waste using enzymes resulted in greenhouse gas impacts 2–18 times lower than sulfuric-acid-based processes for cotton and coconut feedstocks differences largely attributable to reduced electricity use.^145^ Similarly, Espinosa *et al.*^146^ showed a 37% energy reduction by applying extrusion post-hydrolysis, while Rol *et al.*^147^ reported a drop in energy consumption for CNF production *via* seven-pass extrusion from >15 000 kWh t^−1^ down to ∼5000 kWh t^−1^ when enzymes were applied. However, precise energy savings remain underreported, and comparisons must consider variations in nanocellulose modification rates and particle dimensions.

Solvent-free nitration methods and ionic liquid-based nitration are emerging as cutting-edge, sustainable alternatives to traditional nitration techniques, which often involve hazardous solvents. These approaches minimize environmental impact by reducing or eliminating the use of volatile organic compounds and potentially harmful reagents. Solvent-free nitration entirely eliminates traditional solvents, enabling direct interaction between reactants and nitrating agents often at low temperatures or with catalytic assistance. This approach bypasses solvent purification and disposal, reducing waste, cost, and solvent-related side reactions, while sometimes improving yields and product purity.^7^ For instance, the solvent-free nitration of toluene with NO_2_ in the presence of molecular oxygen over an immobilized AlCl_3_–SiO_2_ catalyst at just 35 °C, achieving 86.5% conversion and 100% selectivity to mononitrotoluene with excellent catalyst stability.^7^

In another study, the practical application of saccharin-derived reagents in mechanochemical electrophilic nitration, utilizing vibratory ball milling under LAG (Liquid-Assisted Grinding) conditions to efficiently functionalize a wide array of alcohols and arenes was reported.^148^ This method maintains the traditional electrophilic aromatic nitration mechanism through *in situ* nitronium/nitryl species while eliminating dangerous mixed acids, organic solvents, and lengthy purification processes. Sc(OTf)_3_ was identified as the most effective catalyst for alcohol nitration, producing the nitrate ester with a yield of 90%. This approach offers a recyclable organic nitrating agent, in contrast to traditional methods dependent on H_2_SO_4_/HNO_3_, significantly minimizing solvent and acid waste, enhancing atom economy, and streamlining safety management.

A greener method for producing cellulose nanocrystals (CNCs) from microcrystalline cellulose (MCC) *via* the synergistic combination of an ionic liquid (IL) pretreatment and recyclable solid acid hydrolysis under mild conditions was conducted. In the first stage, MCC is swollen in an IL water mixture (cellulose: IL ratio of 1 : 4, 2% water content) at 45 °C for 30 minutes with ultrasonic assistance.^149^ Subsequently, solid acid hydrolysis is performed at 45 °C for 5 h using a solid acid load of 45 wt% relative to cellulose, yielding rod-like CNCs with lengths of approximately 300 ± 100 nm and widths of 20 ± 10 nm with high thermal stability. The solid acid catalyst can be recovered and reused, to enhance the sustainability profile of the process.

A homogeneous esterification approach to nitrocellulose (NC) synthesis using the ionic liquid [Bmim]Cl as both solvent and reaction medium was introduced. This process enables direct, uniform nitration of cellulose with nitric acid, avoiding the traditional sulfuric–nitric mixed acid system. NC with 12.62 wt% nitrogen was obtained in just 15 minutes.^150^ The resulting material displayed a distinctive 3D honeycomb morphology with 200–300 nm pores, uniform nitrogen distribution, and a low polydispersity index (PDI = 1.55). Performance testing revealed a 40.5% increase in burning rate, 16.4% lower activation energy, and a 141 J per g^−1^ boost in heat release *versus* commercial NC (NC 12.6%).^150^ Compared to conventional methods, this process avoids sulfuric acid, reduces acid waste and by-products (*e.g.*, sulfate esters), and eliminates complex stabilization treatments. Both solvent-free and IL-based methods exemplify green chemistry principles by drastically reducing or eliminating solvent use, minimizing waste, and improving laboratory and industrial safety. Additionally, the recyclability of catalysts and ionic liquids promotes resource conservation and sustainable process design. These innovations ensure that the production processes for cellulose-based polymers adhere to the “design for waste prevention” ethos of green chemistry.

Energy efficiency is another critical focus in the synthesis of nanocellulose-based polymers. Traditional mechanical treatments for producing CNFs were energy-intensive, but modern methods now integrate enzymatic pretreatments or mild chemical processes to reduce energy demands.^151^ For CNC, process optimization has shortened reaction times and lowered energy input during hydrolysis, without sacrificing yield or quality. BNC production is inherently energy-efficient, as microbial fermentation occurs under mild conditions, and its scalability can be enhanced by coupling fermentation systems with renewable energy sources.

By integrating green chemistry principles into the synthesis of CNFs, CNCs, and BNC, the production of nanocellulose-based nitrated polymers demonstrates a commitment to sustainability without compromising functionality. These advancements pave the way for greener propellant technologies, reducing the environmental footprint of high-energy materials while maintaining their critical performance characteristics. As the field continues to evolve, further adherence to green chemistry principles will enable even more efficient, safe, and sustainable production processes.

#### 4.4.1. Reduced use of harmful solvents and reagents

The production of CNFs, CNCs, and BNC, increasingly emphasizes the reduction of harmful solvents and reagents to mitigate environmental impact and enhance sustainability. Traditional chemical processes in the production of energetic materials, including the nitration of nanocellulose, often rely on hazardous substances that pose risks to human health and the environment. By adopting green chemistry principles, such as the development of safer reagents and solvent recovery systems, the production of these materials aligns with global efforts to create more sustainable and eco-friendly propellants.

In line with the green chemistry principle of using safer solvents and reagents, researchers have worked to reduce or replace hazardous chemicals in the nitration of nanocellulose. Innovations include optimizing acid concentrations, employing less toxic nitration agents, and designing closed-loop systems that capture and recycle chemicals, reducing the potential for environmental contamination. These approaches enhance the sustainability profile of nanocellulose-based nitrated polymers while maintaining the high-performance characteristics required for propellant applications.

For CNCs production, the acid hydrolysis process has historically relied on strong mineral acids such as sulfuric or hydrochloric acid, which can generate significant chemical waste and pose environmental hazards.^51^ Recent innovations, however, focus on reducing acid concentrations and optimizing reaction conditions to minimize waste and improve efficiency. Additionally, alternative hydrolysis methods, such as enzymatic or mild acid processes, are being explored to further decrease reliance on harsh reagents. For instance, several mono- and poly-carboxylic organic acids have demonstrated potential for cellulose nanocrystal (CNC) extraction, including citric acid (CA), acetic acid (AA), maleic acid (MA), formic acid (FA), and oxalic acid (OA).^152^ These organic acids offer a greener alternative to traditional mineral acid hydrolysis for CNC production.

In the case of CNFs production, chemical pretreatments often involve reagents such as TEMPO (2,2,6,6-tetramethylpiperidine-1-oxyl) or alkalis, which can be harmful if not managed properly.^153^ Advances in green pretreatment techniques, including enzymatic methods and mechanical refining, have reduced the need for such chemicals, lowering the overall environmental impact. Comparative analysis of cellulose nanofibrils (CNFs) produced through mechanical and enzymatic hydrolysis pretreatments reveals minimal differences in aspect ratio between the two methods. However, enzymatic pretreatment generates CNFs with significantly greater variation in fractal dimension, suggesting this approach creates more heterogeneous nanostructural morphologies.^153^ Furthermore, the integration of safer solvents, such as water-based systems, eliminates the use of organic solvents, which are commonly associated with traditional polymer processing. These improvements not only reduce chemical hazards but also align with efforts to develop cleaner and more sustainable production pathways.

BNC production offers a distinct advantage in this context, as it avoids the need for harmful solvents or chemical pretreatments altogether. BNC is synthesized by microorganisms in aqueous media under mild conditions, often using simple sugars or industrial byproducts as feedstocks. The absence of harsh chemicals in its synthesis significantly reduces environmental risks, making it one of the most sustainable options for producing cellulose-based nitrated polymers. Efforts to optimize the fermentation process further enhance its environmental profile, with minimal chemical inputs and reduced waste generation.

During the nitration of nanocellulose-based materials to create energetic polymers, the use of harmful nitrating agents, such as concentrated nitric and sulfuric acids, is a critical consideration. Advances in reaction optimization, such as employing lower acid concentrations, shorter reaction times, and controlled reaction conditions, have contributed to safer and more efficient nitration processes. Additionally, the development of closed systems to recover and recycle nitration reagents minimizes chemical waste and reduces the overall environmental impact of the production process.

By prioritizing the reduced use of harmful solvents and reagents, the production of CNFs, CNCs, and BNC demonstrates a commitment to sustainability and environmental stewardship. These advancements not only minimize the ecological footprint of cellulose-based nitrated polymers but also enhance their viability as a greener alternative to traditional materials in propellant applications. As the demand for sustainable energetic materials grows, further innovation in reagent efficiency and solvent substitution will be essential to achieving the dual goals of high performance and environmental responsibility.

## 5. Future directions and challenges

The environmental impact and sustainability of nanocellulose-based nitrated polymers derived from CNFs, CNCs, and BNC, offer significant promise in modern propellant technologies. However, several challenges must be addressed to fully realize their potential. Future research and development should focus on enhancing the environmental profile of these nanomaterials while maintaining the high performance required for propellant applications. Key areas of focus include developing greener nanocellulose-based nitrated polymers, improving production processes, balancing performance and environmental priorities, and navigating regulatory landscapes.

A primary area for future innovation is the design of environmentally friendly nitrated nanocellulose polymers with minimal ecological footprint. Current nitration processes often rely on harsh chemicals, such as concentrated nitric and sulfuric acids, which generate hazardous waste. Researchers are exploring alternative nitration methods using milder reagents or enzymatic approaches to reduce chemical hazards. Furthermore, the development of hybrid materials combining cellulose-based nitrates with bio-derived additives or binders may enhance performance while maintaining sustainability. Advanced modeling and material science tools can also help design polymers that optimize energy output with reduced resource consumption.

The scalability of CNFs, CNCs, and BNC production remains a key challenge, particularly in terms of resource and energy efficiency. Addressing this requires continued innovation in production methods, such as adopting enzymatic pretreatments, optimizing acid recovery systems, and integrating renewable energy sources into manufacturing processes. For CNCs, alternative hydrolysis methods that reduce acid usage and waste are being developed, while CNFs production benefits from techniques that minimize mechanical energy input. BNC synthesis can be further optimized by leveraging industrial waste streams as feedstocks, enhancing both economic and environmental benefits.

One of the most significant challenges lies in balancing the performance requirements of propellants with the environmental benefits of cellulose-based materials. While CNFs, CNCs, and BNC offer competitive energy densities and combustion properties, achieving performance parity with traditional synthetic materials often involves trade-offs. Researchers must identify ways to enhance the energetic properties of these polymers without compromising their biodegradability or renewability. Tailoring structural properties through advanced processing techniques and blending nanocellulose-based materials with other sustainable additives may provide a pathway to achieving this balance.

The adoption of nanocellulose-based nitrated polymers also faces regulatory hurdles, particularly in the context of safety, performance standards, and environmental compliance. Existing regulations for energetic materials may need to be updated to account for the unique properties and environmental benefits of nanocellulose-based alternatives. Proactive engagement with regulatory bodies can facilitate the development of standards that recognize the reduced ecological impact of these materials. At the same time, policy incentives and certifications for green technologies could create new opportunities for nanocellulose-based nitrated polymers to gain wider acceptance in the propellant industry.

The path forward for nanocellulose-based nitrated polymers in propellants requires addressing both technical and systemic challenges to enhance their environmental and economic viability. By focusing on greener synthesis methods, improving production efficiency, balancing performance with sustainability, and navigating regulatory landscapes, CNFs, CNCs, and BNC have the potential to redefine propellant technologies. With continued research and innovation, these nanomaterials can contribute to a future where high-performance energetic materials align with global sustainability goals.

## 6. Conclusions

The environmental impact and sustainability of nanocellulose-based nitrated polymers, including CNFs, CNCs, and BNC, present a transformative opportunity for propellant technologies. These nanomaterials offer the dual benefits of renewable sourcing and reduced environmental burden while maintaining potential for high performance. Key findings underscore the importance of innovations in greener synthesis methods, resource-efficient production processes, and the balance between performance and sustainability. Additionally, the distinct advantages of nanocellulose-based nitrated polymers align with global efforts to mitigate the environmental impacts of energetic materials.

Considering environmental impact and sustainability in propellant formulations is no longer optional but essential in meeting contemporary challenges such as climate change and resource depletion. Nanocellulose-based nitrated polymers exemplify how renewable materials can replace traditional, environmentally damaging alternatives, thus supporting a transition toward greener propellants. However, achieving widespread adoption requires continued advancements in production technologies, performance optimization, and regulatory frameworks.

Future research should prioritize the development of safer nitration methods, further optimization of energy and nanomaterial efficiency, and comprehensive life cycle assessments to quantify environmental benefits. Collaborative efforts between academia, industry, and regulatory bodies are crucial to establish these nanomaterials as viable, sustainable alternatives. By addressing these challenges, nanocellulose-based nitrated polymers can play a pivotal role in shaping the future of sustainable propellant technologies.

## Author contributions

The manuscript was written through the contributions of all authors. N. F. A. A.: writing original draft preparation, conceptualization, literature search, visualization. M. N. F. N.: conceptualization, validation, reviewing. A. S.: validation. W. M. Z. W. Y.: reviewing, editing, validation, supervision. All authors have given approval to the final version of the manuscript.

## Conflicts of interest

The authors declare no conflicts to declare.

## Data Availability

The authors confirm that the data supporting the findings of this study are available within the article.
